# The ING1a Tumor Suppressor Regulates Endocytosis to Induce Cellular Senescence Via the Rb-E2F Pathway

**DOI:** 10.1371/journal.pbio.1001502

**Published:** 2013-03-05

**Authors:** Uma Karthika Rajarajacholan, Subhash Thalappilly, Karl Riabowol

**Affiliations:** 1 Department of Biochemistry and Molecular Biology, University of Calgary, Calgary, Alberta, Canada; 2 Department of Oncology, Faculty of Medicine, University of Calgary, Calgary, Alberta, Canada; Lawrence Berkeley Laboratory, United States of America

## Abstract

The INhibitor of Growth (ING) proteins act as type II tumor suppressors and epigenetic regulators, being stoichiometric members of histone acetyltransferase and histone deacetylase complexes. Expression of the alternatively spliced ING1a tumor suppressor increases >10-fold during replicative senescence. ING1a overexpression inhibits growth; induces a large flattened cell morphology and the expression of senescence-associated β-galactosidase; increases Rb, p16, and cyclin D1 levels; and results in the accumulation of senescence-associated heterochromatic foci. Here we identify ING1a-regulated genes and find that ING1a induces the expression of a disproportionate number of genes whose products encode proteins involved in endocytosis. Intersectin 2 (ITSN2) is most affected by ING1a, being rapidly induced >25-fold. Overexpression of ITSN2 independently induces expression of the p16 and p57^KIP2^ cyclin-dependent kinase inhibitors, which act to block Rb inactivation, acting as downstream effectors of ING1a. ITSN2 is also induced in normally senescing cells, consistent with elevated levels of ING1a inducing ITSN2 as part of a normal senescence program. Inhibition of endocytosis or altering the stoichiometry of endosome components such as Rab family members similarly induces senescence. Knockdown of ITSN2 also blocks the ability of ING1a to induce a senescent phenotype, confirming that ITSN2 is a major transducer of ING1a-induced senescence signaling. These data identify a pathway by which ING1a induces senescence and indicate that altered endocytosis activates the Rb pathway, subsequently effecting a senescent phenotype.

## Abstract 2

An age-associated isoform of ING1, ING1a, induces cell senescence by altering endocytosis, subsequently activating the retinoblastoma tumor suppressor.

## Author Summary

Alternative splicing of several genes including the p16 and p53 tumor suppressors has been reported to increase during replicative senescence of normal diploid cells, but the biological functions of most alternative transcripts are unknown. We have found that a splicing product of the *ING1* epigenetic regulator, ING1a, also increases during senescence; moreover, forced expression of ING1a at these levels in otherwise growth-competent cells can induce senescence. In this study we have determined that a major mechanism by which ING1a induces senescence is through inhibiting endocytosis; this subsequently activates the retinoblastoma (Rb) tumor suppressor pathway by increasing Rb levels and preventing its inactivation through multiple mechanisms. Our study also establishes a link between endocytosis and oxidative stress and suggests that multiple mechanisms that induce cellular senescence may do so by inhibiting normal endocytic processes, thereby affecting normal signal transduction pathways including those mitogenic pathways required for cell growth.

## Introduction

Cellular senescence was first described as a consequence of the limited replicative capacity of human diploid fibroblasts by Hayflick in the early 1960s [1]. It was later characterized as an intrinsic tumor-suppressive mechanism that acts to limit the proliferative capacity of precancerous cells. Replicative senescence is triggered by telomere erosion [2], the loss of TTAGGG nucleotide repeats that occurs as a consequence of the end replication problem of linear chromosomes, where DNA polymerase is unable to synthesize the extreme termini of lagging DNA strands [2],[3]. Senescence, resulting in permanent cell cycle arrest, can also be induced independent of telomere loss as a consequence of various forms of stress, including oncogenic [4] and oxidative stress [5],[6], and has been referred to as stress-induced premature senescence, or SIPS [7]. Markers for senescence include senescence-associated β-galactosidase activity (SA-β-gal) [8]; formation of senescence-associated heterochromatic foci (SAHF) [9]; accumulation of lipofuscins [10]; changes in nuclear morphology [11]; increased p16INK4a [12], cyclin D1 [13], and cyclin D2 [14] levels; loss of gene inducibility [15]; and hyperactivation of the pRb [16] and p53 [17] tumor suppressors. In addition, alternative splicing of mRNAs from diverse genes [18] including those encoding proteins that affect chromatin structure such as p53 [19], p16 [20], Pot-1 [21], lamin A [22], and ING1a [23] has been reported to increase during replicative senescence, and the telomere-initiated stress signal has been implicated in promoting the production of alternative splice products [22].

The INhibitor of Growth (ING) family consists of five genes (ING1–5) encoding multiple splice products [24],[25]. All ING proteins contain plant homeodomains (PHDs) through which they bind the histone H3 epigenetic mark H3K4Me3 [26]–[28], thus serving as epigenetic readers. They are also stoichometric members of histone acetyltransferase (HAT) and histone deacetylase (HDAC) complexes [29], directing their activities to adjacent histone amino acid residues to alter chromatin structure [30] and affect transcription [31]. The ING proteins also contain a sequence unique in the human proteome called the lamin interacting domain through which they physically interact with lamin A [32], suggesting that altered localization and levels of the INGs may contribute to the Hutchinson Gilford Progeria Syndrome (HGPS) form of premature aging. HGPS cells show altered chromatin conformation and nuclear membrane structure that is caused by alternative splicing of the lamin A gene and subsequent production of a truncated form of lamin A called progerin [33]. The INGs function as type II tumor suppressors, being frequently down-regulated or mislocalized in different tumor types [34]–[37], and murine knockout models of ING1 show development of B cell lymphoma independent of p53 status [38], although ING1 protein can increase p53 levels through effects upon p53 polyubiquitination [39].

The ING1 gene encodes four variants, with p33ING1b and p47ING1a being the best characterized and predominant isoforms [23],[37],[40]. Overexpression of the major isoform, ING1b, initially induces features of stress-induced senescence such as SA-β-gal activity, increased expression of p16 and growth arrest [41]–[43], and culminates in cells acquiring pyknotic nuclei and undergoing apoptosis [44]. In contrast, overexpression of ING1a blocks cell growth in a state that resembles replicative senescence by a number of criteria including high SA-β-gal activity, presence of SAHF, increased cell size, altered nuclear morphology, increased expression of p16 and Rb, and growth arrest [23]. Furthermore, as cells undergo replicative senescence, the ratio of ING1a:ING1b increases by ∼30-fold [23], and knocking down ING1 [45] or ING2 [46] in senescing fibroblasts significantly increases their replicative life span in culture, suggesting roles for the INGs in transducing telomere-initiated senescence signaling. Despite these observations linking ING1a to the induction of senescence, its role in replicative senescence and the mechanism by which it induces SIPS have yet to be determined.

Here we ask what genes are regulated by altered ING1a levels in order to better understand how ING1a functions in senescence. We find that ING1a affects growth factor receptor internalization by transcriptional up-regulation of a group of genes whose products affect endocytosis, subsequently activating the retinoblastoma tumor suppressor pathway. Furthermore, inhibition of endocytosis in young fibroblasts by several methods results in phenotypes resembling senescence, supporting the idea that alterations in signal transduction, at least partly as a consequence of ING1 alternative splicing, contribute to establishing the senescence phenotype.

## Results

### ING1a Induces the Expression of Endocytic Genes

**Figure 1:**
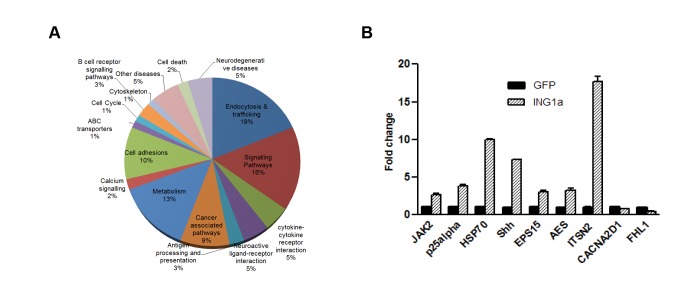
Gene expression in response to ING1a. (A) Functional categorization of 242 genes reproducibly up-regulated in response to ING1a overexpression. Thirty-five percent of all up-regulated genes and 40% of those induced >2.5-fold had functions in endocytosis, trafficking, and associated signaling pathways. (B) Quantitative PCR (qPCR) validation of mRNA levels of a representative set of genes up- and down-regulated in ING1a overexpressing cells. Bars represent standard deviations of three independent trials (*p*<0.05).

To investigate how ING1a induced SIPS when overexpressed and to elucidate its role in replicative senescence, we identified genes that are differentially regulated by ING1a using microarray-based analysis in human diploid fibroblasts. Hs68 cells were infected with replication-deficient adenoviral vectors encoding ING1a and GFP under separate promoters (Ad-ING1a) or control virus encoding GFP (Ad-GFP) alone, and grown for 48 h. The analysis identified 242 up-regulated and 172 down-regulated genes that showed significantly different expression levels upon ING1a overexpression (Tables S1 and S2). Figure 1A shows the functional categories of the up-regulated genes as estimated by various pathway analyses. A list of genes that were reproducibly altered by mean fold changes greater than ±2.5-fold is shown in Table 1. Among the genes that exhibited significant differences in expression, >40% were known to function in endocytosis, vesicular trafficking, or related signaling (marked with asterisks in Table 1). A subset of these genes was analyzed by qPCR to confirm the array results, and all the genes tested validated the microarray experiment (Figure 1B).

**Table 1:** Genes (A) up-regulated or (B) down-regulated at least 2.5-fold in response to ING1a overexpression in primary Hs68 fibroblasts.

(A) Gene Name	Fold Increase
SH3 domain protein 1B (Intersectin 2) (ITSN2)*	27.65*
Janus kinase 2 (a protein tyrosine kinase) (JAK2)*	11.27*
Hypothetical protein	8.32
Heat shock 70 KDa protein 1 (HSP70)	7.31
Glutaryl-coenzyme A dehydrogenase (GCDH)	4.89
Amino terminal enhancer of split (AES)	4.77
Sonic hedgehog (Drosophila) homolog (SHH)	4.68
Glioma tumor suppressor candidate region gene 1 (GLTSCR1)	4.6
Pancreatic lipase-related protein 2 (TNN13K)	4.16
Putative protein-tyrosine kinase*	4.12*
Epidermal growth factor receptor substrate 15 (EPS15)*	3.98*
Mitogen-activated protein kinase 7 (MAPK7)*	3.86*
FLT4*	3.76*
Hypothetical protein DKFZp434C0923	2.84
Brain-specific protein p25 alpha (p25 alpha)	2.61
Adaptor related protein complex AP4 mu4 subunit (AP4)*	2.53*

(B) Gene Name	Fold Increase
Calcium channel, voltage dependent, alpha 2/delta subunit 1	−13.03
Endothelial cell growth factor 1 (platelet derived)	−10.63
Golgi SNAP receptor complex member 2	−4.43
Protein (peptidyl-prolyl cis/trans isomerase) NIMA-interacting 1	−3.17
Aldehyde dehydrogenase 1, soluble	−2.8
Four and a half LIM domains 1	−2.07

Cells expressing GFP were used as a negative control. Seven of the 17 most highly induced genes (>40%) highlighted by asterisks have characterized functions in endocytosis, signaling, and trafficking.

The gene showing the largest fold change in response to ING1a expression, was intersectin 2 (ITSN2), a key component of endocytosis. ITSN2 is a 180 kDa multidomain adaptor protein, containing two Eps homology (EH) domains, a coiled coil (CC) domain, and five Src homology 3 (SH3) domains. Alternative splicing generates a longer isoform that has an additional Dbl homology (DH) domain, a pleckstrin homology (PH) domain, and a C2 domain [47]–[49]. ITSN2 facilitates the assembly of endocytic proteins for the formation of clathrin pits during clathrin-mediated endocytosis of growth factor receptors. It interacts with epsin, a clathrin pit component, and with AP2, a clathrin adaptor complex, through its EH domains [50],[51], and binds to dynamin and synaptojanin, two proteins needed for the pinching off of clathrin vesicles from the membrane surface, through its SH3 domains [49],[52]. ITSN2 forms heterodimers with EPS15, an essential component of the endocytic pathway [53], through its CC domain. Interestingly, we found that EPS15 expression was also altered by ING1a in our microarray (Table 1) and RT-PCR analyses (Figure 1). It has previously been reported that overexpression of ITSN2 inhibits transferrin (TR) and epidermal growth factor receptor (EGFR) internalization and blocks clathrin-mediated endocytosis [54]–[56]. Intersectin proteins may do this by virtue of their five SH3 domains, since overexpression of the SH3 domain of ITSN affected its interaction with dynamin and also inhibited endocytosis by causing the formation of constricted clathrin-coated pits [57]. To study the effect of ITSN2 expression in fibroblasts, we ectopically expressed ITSN2 in Hs68 cells and checked for EGF receptor internalization. We found that cells overexpressing ITSN2 had reduced EGFR uptake after 10 min of EGF stimulation (Figure S1). The second most highly ING1a-regulated gene was JAK2 (Table 1), the Janus kinase that regulates the internalization and turnover of several receptors including the growth hormone receptor [58] and the interleukin-5 receptor [59]. The fact that ITSN2, JAK2, and EPS15, as well as other proteins that affect endocytosis, were selectively regulated by ING1a suggested that ING1a might affect endocytosis, a process that regulates cell signaling and growth in response to extracellular stimuli.

### ING1a Regulates Endocytosis

**Figure 2:**
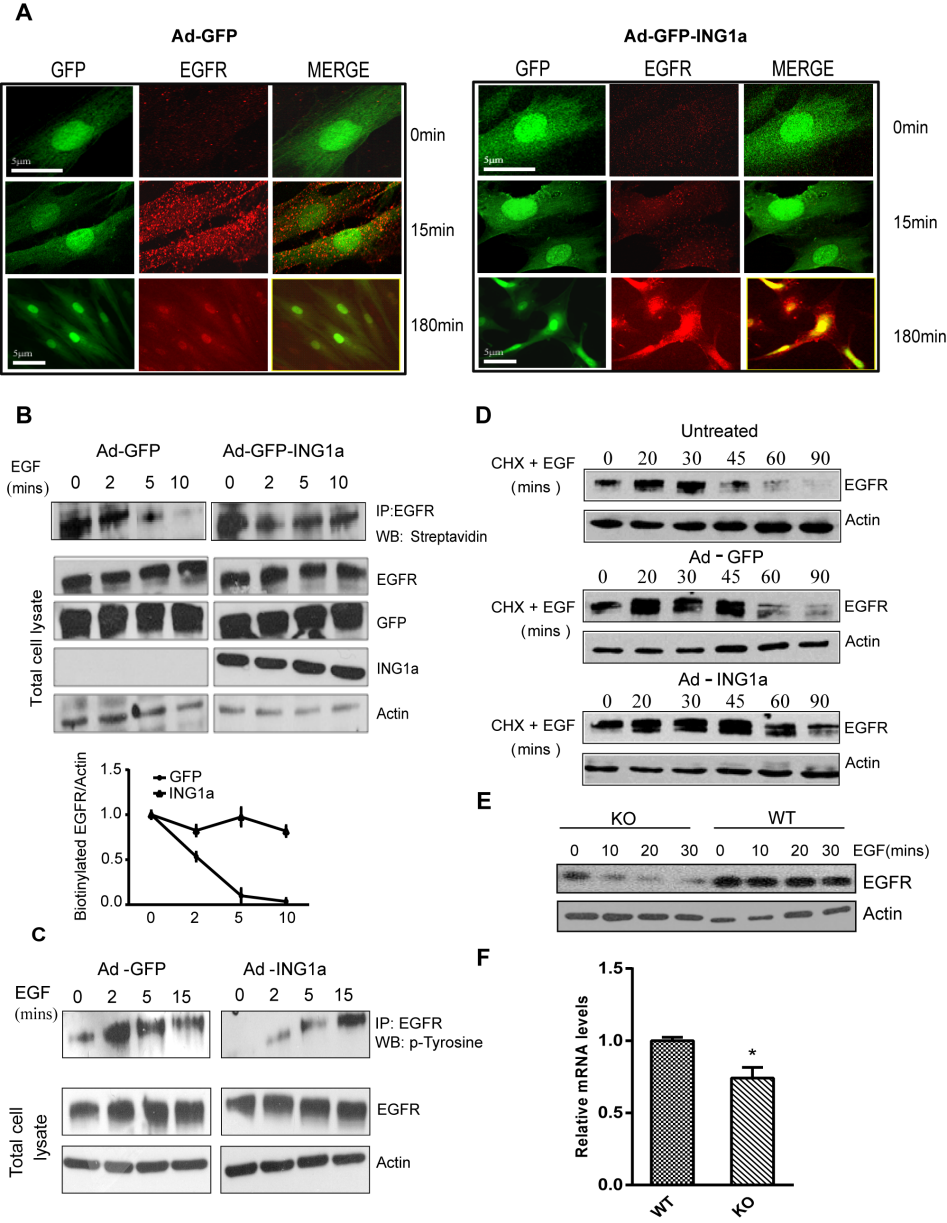
Regulation of endocytosis by ING1a. (A) Cells infected with adenovirus-GFP or adenovirus-GFP-ING1a (with GFP and ING1a expressed under separate promoters) were serum-deprived for 12 h, stimulated with 100 ng/ml EGF, and fixed 15 or 180 min later. Indirect immunofluorescence for EGFR showed reduced amounts of internalized EGFR in cells expressing ING1a at early time points but persistence of the EGFR at later times. All cells were treated with 10 µg/ml of cycloheximide to inhibit de novo protein synthesis. We took 0- and 15-min images using 63× objectives, while 180 min were imaged at 40× magnification. (B) Biotin internalization assay. Ad-GFP-ING1a and Ad-GFP-expressing cells were serum-starved and EGF-stimulated for the indicated times. Total cell surface proteins were biotinylated, and EGFR was immunoprecipiatated. Biotinylated cell surface EGFR at the indicated time points was detected using streptavidin-HRP. The graph shows the rate of EGFR internalization in GFP- and ING1a-expressing cells as estimated by scanning densitometry. (C) A431 cells infected with adenovirus-GFP/ING1a were serum-starved and checked for tyrosine phosphorylation of the immunoprecipiated EGFR upon EGF stimulation for the indicated time-points. (D) Cells left untreated or expressing GFP or GFP plus ING1a for 48 h were serum-starved overnight (12 h) and stimulated with 100 ng/ml EGF for the indicated times. 100 µg/ml of cycloheximide was used to inhibit protein synthesis. Cells were lysed and levels of EGFR were analyzed by western blotting. Actin was used as a loading control. (E) Wild-type or ING1 knockout MEFs were serum-starved overnight, treated with 10 µg/ml of cycloheximide for 20 min, stimulated with 100 ng/ml EGF and were harvested at the indicated times. Proteins were resolved by SDS-PAGE and blotted with α-EGFR antibody and α-actin as loading control. (F) mRNA levels of Ese2, the ITSN2 mouse homologue in MEF WT and ING^−/−^ cells. RNA was isolated from these cells, and qRT-PCR was performed on three independent replicates. The gene expression levels were normalized to GAPDH (*p*<0.05).

In order to test the hypothesis that ING1a was inducing features of cellular senescence through its effects on endocytosis, we studied the effect of ING1a expression on endocytosis of the EGF receptor, since it is the best characterized receptor in terms of internalization and trafficking [60]. EGFR uptake and retention were analysed in ING1a-expressing Hs68 cells at various time points after EGF stimulation. As shown in Figure 2A, immunofluorescence analysis showed that control cells had more EGFR puncta (endosomes) after 15 min of EGF stimulation compared to ING1a-expressing cells. Furthermore we found that EGFR staining was retained in ING1a-expressing cells at later time points (3 h of EGF stimulation), while they were absent in the control cells. These observations suggested that ING1a expression delayed both the internalization of EGF receptor as well as its degradation. Similar pulse chase experiments were also carried out to study the colocalization of EGFR with Rab5 (an early endosome marker) and Rab7 (a late endosome marker) in control and ING1a-expressing fibroblasts, and in all the cases we found that ING1a-expressing cells showed delayed trafficking of EGF receptor (unpublished data).

To further confirm the difference in EGFR internalization, surface biotinylation assays were carried out in A431 cells, which express high levels of endogenous EGFR. Consistent with the immunofluorescence results, ING1a-expressing A431 cells retained EGFR on the cell surface for a longer time compared to GFP-expressing cells (Figure 2B). We also checked the tyrosine phosphorylation status of EGF receptor to see if there was a difference in the activation of the receptor, prior to internalization, in A431 cells. We found delayed tyrosine phosphorylation on EGFR immunoprecipitated from ING1a-expressing A431 cells. Control cells had tyrosine-phosphorylated EGFR starting within 2 min of EGF stimulation, while in ING1a-expressing cells, a significant amount of phosphorylation was visible only after 15 min (Figure 2C). These results confirmed that EGFR internalization is significantly delayed when ING1a was overexpressed. To study the degradation of EGF receptor, ING1a-expressing cells were treated with cycloheximide, harvested at different time points after EGF stimulation, and were analyzed by western blotting for EGFR levels. ING1a-expressing cells retained significant levels of EGFR even 90 min after EGF stimulation, while in the control cells, most EGFR was degraded by 60 min (Figure 2D). This result corroborated the observation of immunofluorescence (Figure 2A) and confirmed that EGFR degradation was delayed when ING1a was overexpressed.

While these results indicate that ING1a inhibited endocytosis and processing of the EGF receptor, these assays all relied on ING1a overexpression and were thus done under supraphysiological levels of ING1a. To verify whether this effect on endocytosis was also mediated by endogenous levels of ING1a, we compared the kinetics of EGF-dependent EGFR degradation in wild-type and in ING1 knockout mouse embryo fibroblasts. As shown in Figure 2E, EGFR levels were lower, and its degradation in ING1^−/−^ cells was more rapid than in the control MEF WT cells. Furthermore, the expression levels of Ese2, the mouse homologue of ITSN2, were significantly reduced in the ING1^−/−^ cells compared to WT MEFs (Figure 2F). These observations confirmed that ING1 is a regulator of ITSN2 expression and has a negative effect on endocytosis. Although the mouse ING1 splice variants are not well characterized, the presence of a murine ING1a isoform, with homology to human ING1a, is predicted based on sequence analysis. We tested for the presence of this ING1a-specific motif by PCR using cDNA obtained from mRNA of MEF WT and ING1 knockout cells. MEF WT cells expressed this region, while ING1 KO cells did not show any expression, confirming that mouse ING1 KO cells lacked this isoform with sequence homology specific for human ING1a (Figure S2).

### Differential Expression of Intersectin 2 in Senescent Cells

**Figure 3:**
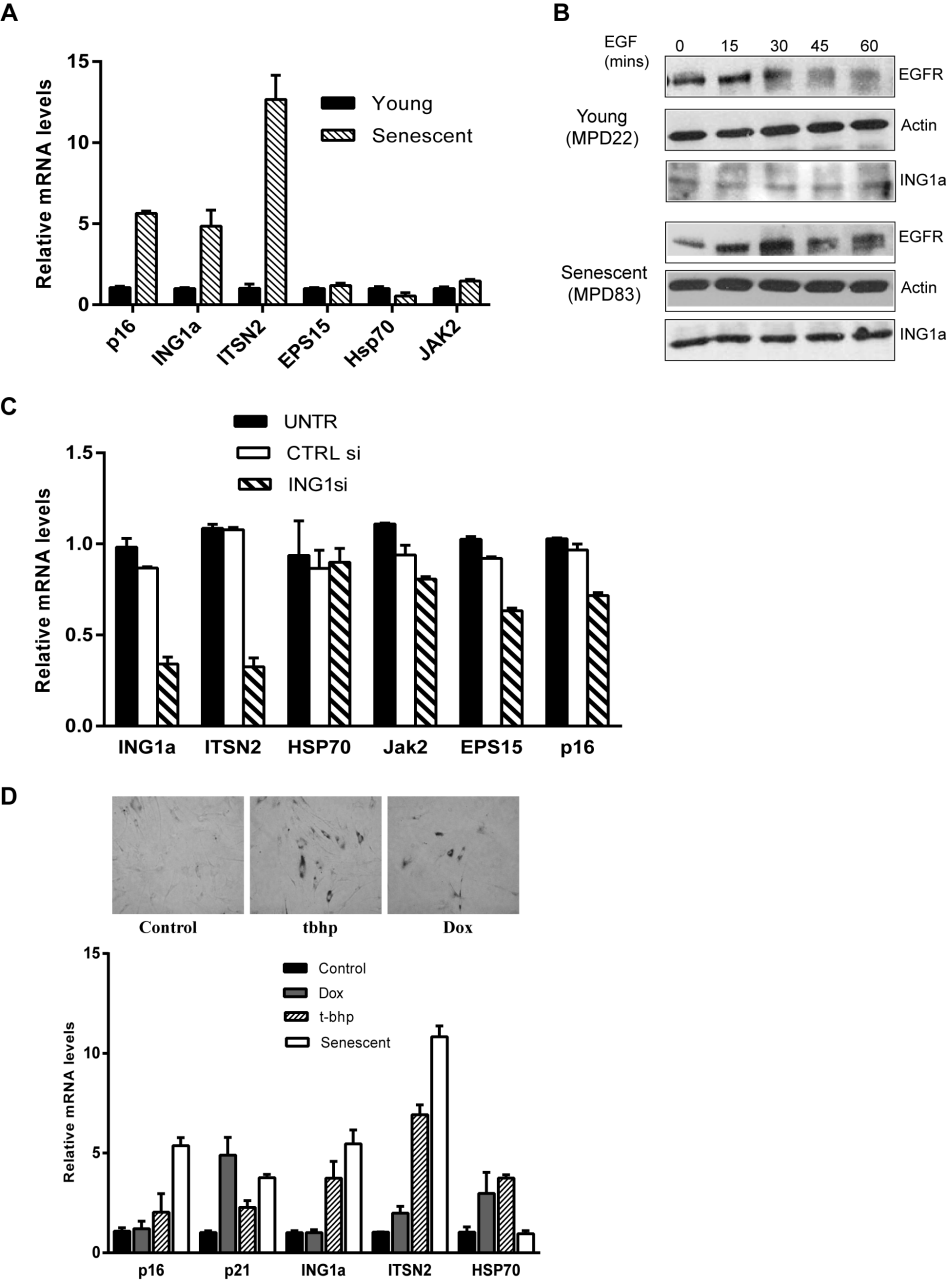
Gene expression levels and kinetics of endocytosis in senescing cells. (A) mRNA levels of ITSN2, EPS15, HSP70, and JAK2 were compared in young and old fibroblasts. Levels were normalized to GAPDH. Levels of p16 and ING1a mRNA serve as controls to confirm the senescent state of cells. (B) EGFR degradation was examined in low passage and senescing cells. The samples were prepared as described previously, and EGFR amounts were estimated by western blotting. EGFR persisted in senescent compared to young cells, and ING1a was expressed at much higher levels in senescent cells as previously reported [23]. (C) mRNA levels of ITSN2 and other endocytic genes in cells knocked down for ING1a using siRNA (*p*<0.05). (D) ING1a and ITSN2 levels in other forms of stress induced premature senescence (SIPS) using doxorubicin and t-butyl hydroperoxide. P16 and p21 are cyclin-dependent kinase inhibitors that serve as senescence markers, and HSP70 as a stress marker. The top panels show SA-β-gal staining as a marker for senescence induction in response to the treatments noted.

Since the expression of ING1a is induced during replicative senescence [23] and we had found that ING1a induced ITSN2 expression, we next examined ITSN2 levels in senescent cells. As shown in Figure 3A, endogenous ITSN2 levels were, indeed, significantly higher in senescent cells compared to low passage young fibroblasts. As we have previously reported, p16 and ING1a levels were up-regulated in senescent cells [23]. However, other genes that were induced by ING1a, such as EPS15, HSP70, and JAK2, did not show significant changes in senescent cells. To check if the higher endogenous levels of ING1a and ITSN2 in senescent cells might correlate with delayed endocytosis during senescence, we compared EGFR degradation at different times after EGF stimulation in young and old Hs68 cells. As shown in Figure 3B, EGFR persisted considerably longer in senescent cells after stimulation with EGF compared to young cells. These data support our hypothesis that, like cells in which ING1a is ectopically expressed, an increase in endogenous ING1a may also contribute significantly to the process of replicative senescence via inhibiting the endocytic pathway.

To further ask whether levels of ING1a, similar to those seen during normal cell senescence, affected ITSN2, we ectopically expressed ING1a to levels comparable to its physiological levels in senescent cells using plasmid transfections. We found that a 2-fold increase in ING1a did not significantly induce ITSN2, while a 5-fold increase in ING1a induced ITSN2 to levels similar to those seen in senescent cells (Figure S3). We next tested if ING1a levels directly regulated ITSN2 induction in senescent cells. We measured the expression of ITSN2 in senescent cells after knocking down ING1a using siRNA. We found that knockdown of ING1a in senescent cells led to significant down-regulation of ITSN2 mRNA levels, further suggesting a role for ING1a in regulating ITSN2 expression (Figure 3C).

To test if ING1a functions in other forms of stress-induced premature senescence (SIPS), we induced senescence using tert-butyl hydroperoxide (chronic oxidative stress) and doxorubicin (DNA damaging agent). While both agents induced SA-β-gal staining, we observed that ING1a levels increased in cells undergoing oxidative stress-induced senescence but not DNA-damage-induced senescence (Figure 3D). ITSN2 levels were also induced by oxidative stress, but not by doxorubicin, consistent with the induction of ITSN2 by ING1a in senescing cells. We further asked whether these forms of senescence also displayed aspects of defective endocytosis. We found that EGF receptor endocytosis was significantly delayed in cells induced to senesce using tert-butyl hydroperoxide. In contrast, the DNA-damaging agent doxorubicin had little effect upon endocytosis of the EGFR (Figure S4).

### Increased Intersectin 2 Expression Precedes the Appearance of Senescence Markers

**Figure 4:**
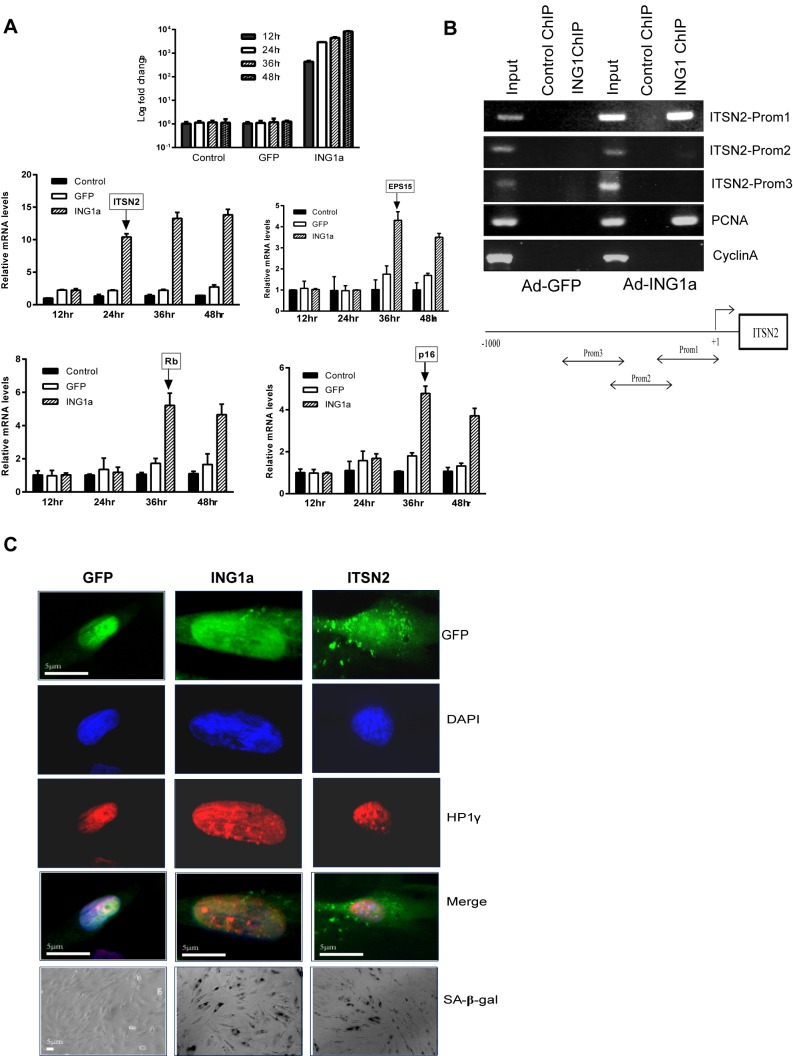
ITSN2 expression precedes the appearance of senescence markers. (A) RNA from uninfected, GFP, or GFP-ING1a-expressing Hs68 cells was isolated at 12, 24, 36, and 48 h post-virus infection. Levels of ING1a mRNA were checked to confirm that ING1a was overexpressed at each of the indicated time points (top most panel). Induction of ITSN2, EPS15, p16, and Rb were checked at these time points by qRT-PCR using gene-specific primers. Arrows indicate the time points at which the induction of expression of each of these genes were noted. (B) Binding of ING1a to the ITSN2 promoter was tested by chromatin immunoprecipitation assay. Binding was not seen in the control GFP-expressing cells. The lower panel is the schematic representation of the location of the primer sequences in the upstream region of ITSN2 that were used in this study. Mouse IgG was used as a nonspecific antibody control. PCNA and Cyclin A promoter regions were used as positive and negative controls for the ChIP assay, respectively. (C) ITSN2 overexpression for 48 h in young fibroblasts resulted in formation of senescent-associated heterochromatin foci, containing the heterochromatin protein 1 (HP1γ), similar to those seen in response to ING1a. Cells also showed significant SA-beta gal staining in response to ING1a and ITSN2.

As noted previously, ING1a induced the expression of both p16 and Rb when ectopically expressed in young fibroblasts [23]. ING1a also induced senescence-associated β-galactosidase staining and cell cycle arrest at the G_0_/G_1_ phase of the cell cycle after about 48 h of ectopic expression [23]. If ITSN2 that is induced by ING1a contributes to the cellular senescence phenotype, we hypothesized that its expression should precede that of the senescence markers associated with ING1a expression. We tested this hypothesis by doing a time course experiment to check the expression levels of p16, Rb, and ITSN2, after ING1a overexpression in young fibroblasts. We found that ING1a levels begin to increase significantly between 12 and 24 h post-infection with Ad-ING1a. ITSN2 levels increased 24 h after infection with Ad-ING1a and reached maximum levels 36 h post-infection. In contrast, mRNA levels of p16 and Rb did not increase until 36 h post-infection (Figure 4A). The other differentially regulated microarray target gene, EPS15, also increased, but only 36 h post-infection. Thus, ING1a induced ITSN2 levels well ahead of Rb and p16, suggesting an upstream, causative role for ITSN2 in mediating the ING1a-initiated senescence signal.

To ask if the transcriptional induction of ITSN2 and EPS15 by ING1 was a direct or indirect effect, we checked whether ING1a binds to the promoters of these genes by chromatin immunoprecipitation using an ING1-specific monoclonal antibody [61]. Although no binding to the EPS15 promoter was seen, we detected binding to a region 200 bp upstream of the ITSN2 gene start site. As shown in Figure 4B, the ING1 antibody but not the control IgG recovered the ITSN2 promoter. These observations support the idea that ING1a drives the expression of ITSN2 by directly binding its promoter, leading to its induction before the appearance of the known senescence markers. The specificity of the antibody used for this assay was confirmed using western blotting (Figure S5).

To confirm the role of ITSN2 in the induction of senescence, we overexpressed ITSN2 in young primary fibroblasts and checked for senescence markers. Ectopic expression of ITSN2 by itself was able to induce SA-heterochromatic foci (SAHF) and SA-beta galactosidase staining in young fibroblasts (Figure 4C). In contrast, ITSN2-expressing cells did not exhibit the enlarged or flattened nuclear and cellular morphology typical of senescent cells and ING1a-expressing cells, suggesting that ITSN2 transduced many, but not all of the ING1a senescence signal and that ITSN2 induction is necessary, but not sufficient for ING1a-induced SIPS.

### Altered Signalling Affects the Rb-E2F Pathway

**Figure 5:**
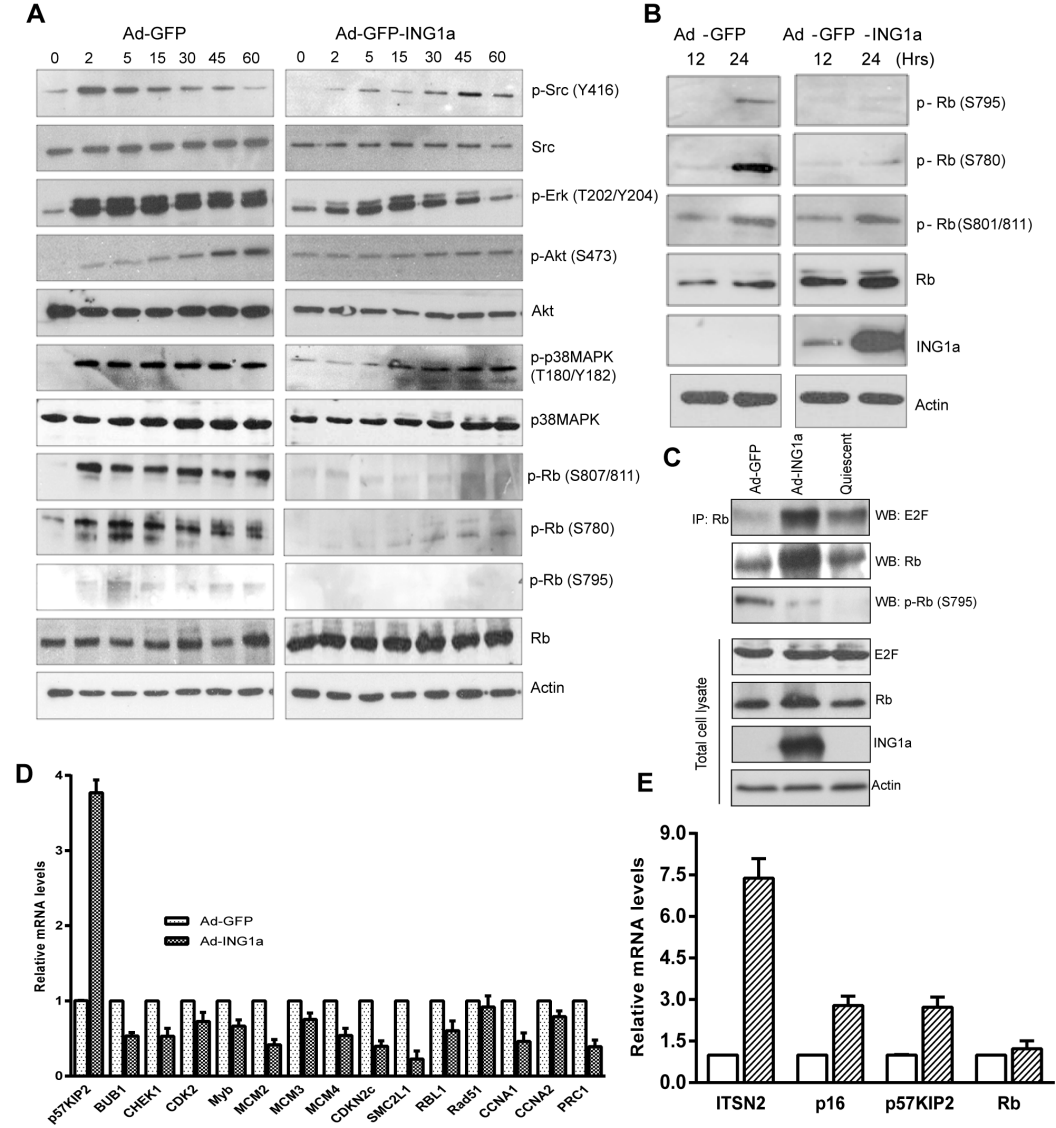
ING1a delays growth factor signaling, delays Rb phosphorylation, and inhibits E2Factivation. (A) Low passage (young) Hs68 fibroblasts (MPD 25) infected with Ad-GFP or Ad-ING1a for 24 h were serum-starved and stimulated with 100 ng/ml of EGF for the indicated time points. Western blots of p-Src, p-Erk, p-Akt, p-38MAPK, and p-Rb were performed to check their activation status as estimated by their phosphorylation. The same lysates were also probed with antibodies for total Src, Akt, p38MAPK, and Rb. Actin was used as the internal loading control. (B) Hs68 cells synchronized by serum starvation for 16 h were released in the presence of Ad-GFP or Ad-ING1a and checked for the phosphorylation status of Rb at indicated time points. (C) Hs68 cells synchronized by serum starvation for 24 h were released in complete medium in the presence of Ad-GFP or Ad-ING1a. Twenty-four hours later, cells were harvested and lysates were immunoprecipiated using anti-Rb antibody. Immunoprecipitates were electrophoresed and blotted with the indicated antibodies. Levels of E2F, Rb, actin, and ING1a were checked in whole cell lysates. (D) Relative mRNA levels of E2F target genes in cells expressing Ad-GFP or Ad-ING1a. The values were normalized to actin (*p*<0.05). (E) Total RNA from cells transfected with ITSN2 was isolated and the expression of p16, p57^KIP2^, and Rb were examined. All values were normalized to levels seen in cells transfected with the same amount of control GFP plasmid, which was also used to monitor and control for transfection efficiency.

To investigate the role of signaling changes associated with altered endocytosis in cells expressing ING1a, we examined the phosphorylation of signaling proteins after EGF stimulation. As noted in Figure 5A, there was a significant delay or attenuation of the phosphorylation of Src (S416), Erk (T202/Y204), p38MAPK (T180/Y182), and Akt (S473) in ING1a-expressing cells compared to control cells. We next examined if changes in growth factor signaling pathways affected the retinoblastoma protein (Rb). Modulation of Rb function by phosphorylation is one of the key mechanisms of senescence induction in cells and mitogenic stimuli alters the phosphorylation status of Rb. Analysis of the Rb phosphorylation sites that inhibit its role as an inhibitor of E2F transcription factor [62]–[64] showed that ING1a expression blocked S807/811 and S795 phosphorylation and strongly inhibited S780 phosphorylation (Figure 5A). These results suggested that, in ING1a-expressing cells, Rb remained tightly bound to E2F, blocking its ability to promote cell proliferation.

We next examined the phosphorylation status of Rb in ING1a-expressing cells during growth in complete medium containing serum. Low passage primary fibroblasts synchronised by serum starvation were released in the presence of Ad-GFP or Ad-ING1a for the indicated time points, and the phosphorylation status of Rb was checked using western blotting with site-specific antibodies. Unlike control cells infected with adenovirus-expressing GFP, which phosphorylated Rb on S780 and S795, ING1a-expressing fibroblasts failed to phosphorylate RB at these residues (Figure 5B). However, under these growth conditions, there was no significant difference in S801/811 phosphorylation. ING1a-expressing cells also expressed significantly higher levels of Rb, consistent with the transcriptional induction of Rb by ING1a [23]. Since hypophosphorylated Rb is the active form that binds and inhibits E2F, we next asked whether the Rb in ING1a-expressing cells physically associated with E2F. Western blot analysis of immunoprecipitated E2F1 in these samples confirmed that E2F bound Rb, avidly in the presence of ING1a compared to the GFP-expressing cells (Figure 5C). Serum-starved quiescent cells were used as a positive control for this experiment. We also noted that higher amounts of Rb protein were immunoprecipitated in ING1a-expressing cells, further confirming the induction of Rb in these cells. As predicted, Rb immunoprecipitated from ING1a-expressing cells was hypophosphorylated at S795 compared to Rb from cells infected with control virus. These observations confirmed that the increased level of Rb in ING1a-expressing cells was maintained in an active, hypophosphorylated state that bound tightly to E2F.

Hypophosphorylated Rb binds E2F to block transcription. Since ING1a-expressing cells showed hypophosphorylated Rb, we measured mRNA levels of a representative number of E2F target genes known to function in different processes including cell cycle progression, DNA synthesis and replication, DNA repair, and checkpoints [65]. As shown in Figure 5D, nearly all the E2F targets investigated were expressed at significantly reduced levels in ING1a-expressing cells. However, one E2F target gene that is a negative regulator of cell cycle progression, p57^KIP2^, was induced in ING1a-expressing cells. Given that p57^KIP2^ is a potent inhibitor of the cyclinE–CDK2 and cyclin D–CDK4 complexes [66] that phosphorylate and inactivate Rb in response to mitogens, these data suggested that the greater amounts of Rb protein expressed in response to ING1a were also maintained in an active state by both p16 and p57^KIP2^ to inhibit cell proliferation and contribute to the induction of senescence. To check if the induction of p57^KIP2^ and p16 in ING1a-expressing cells was mediated through ITSN2, we overexpressed ITSN2 to levels comparable to those seen in untransfected senescent cells and found that ITSN2 was able to independently induce both p16 and p57^KIP2^, but not Rb (Figure 5E), suggesting that the CDK inhibitors were induced as a consequence of ITSN2, while induction of Rb by ING1a occurred through an ITSN2-independent pathway.

### ITSN2 Knockdown Antagonizes ING1a Induced Senescence

**Figure 6:**
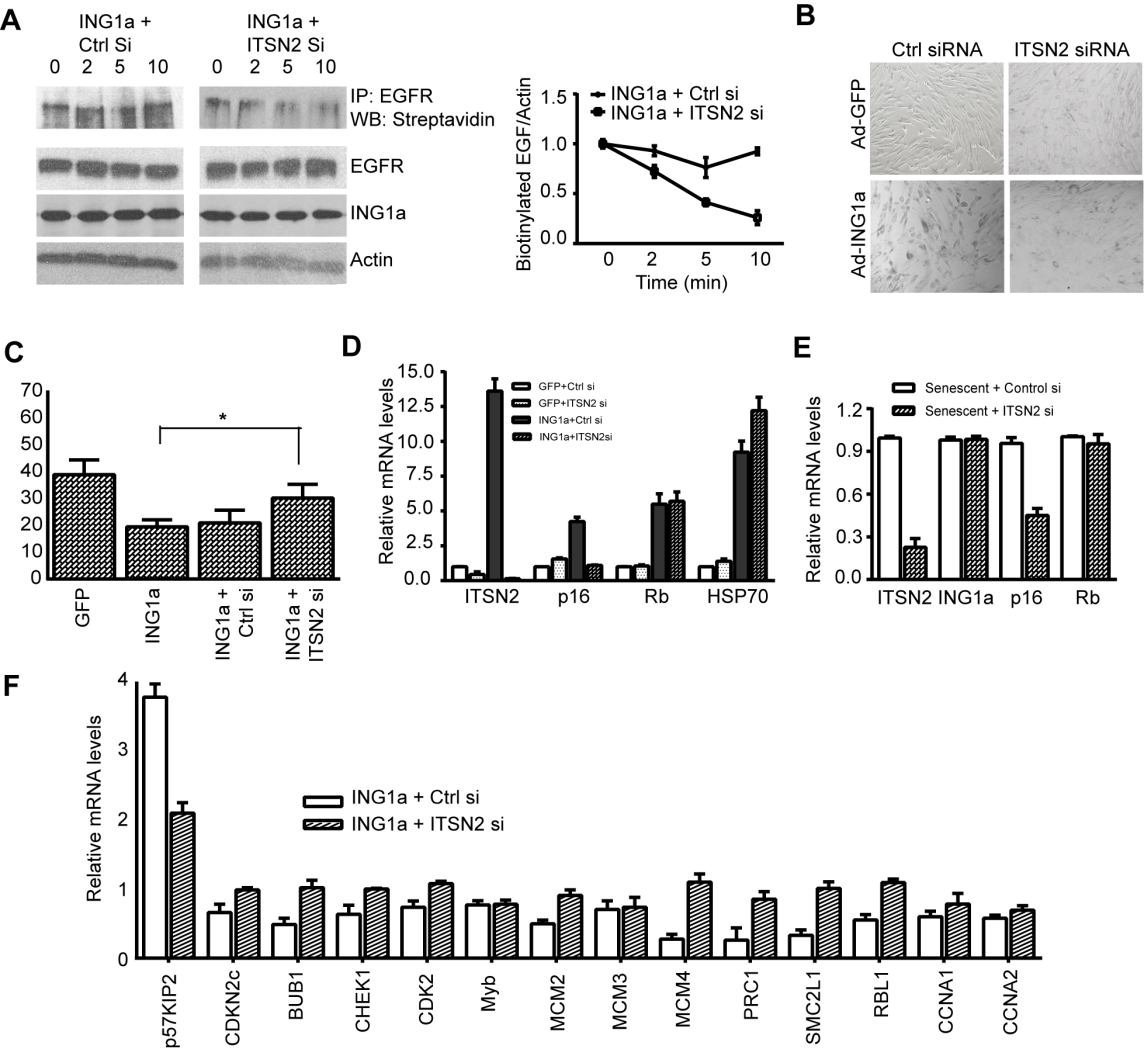
ITSN2 knockdown ameliorates ING1a-induced senescence phenotypes. (A) Biotin internalization assay in A431 cells expressing Ad-ING1a+Control siRNA or ITSN2 siRNA. Cells were serum-starved overnight and stimulated with EGF for the indicated times. Biotinylated cell surface EGFR was quantified using scanning densitometry. Results of three trials are shown in the graph. (B) A β-gal assay was carried out in cells expressing GFP and ING1a after transfection with control or ITSN2 siRNA. (C) Hs68 cells expressing Ad-GFP, Ad-ING1a, or Ad-ING1a together with control siRNA or ITSN2 siRNA were analysed for proliferation using a BrdU incorporation assay. The percentage of cells that incorporated BrdU is presented in the histogram (*p*<0.05). (D) Low passage (young) Hs68 cells were transfected with control siRNA (50 nM) or ITSN2 smartpool siRNA (50 nM). Twenty-four hours later cells were infected with Ad-GFP or Ad-ING1a-expressing viruses, and 48 h later the levels of ITSN2, p16, RB, and HSP70 mRNA were measured by quantitative real-time PCR using gene-specific primers. The mRNA levels were normalized to β-actin levels. (E) Senescent Hs68 cells (MPD80) were transfected with control siRNA or ITSN2 siRNA and checked for the relative levels of the indicated mRNAs using quantitative real-time PCR. The values were normalized to actin. (F) Relative mRNA levels of representative E2F target genes in ING1a-expressing cells transfected with control siRNA or ITSN2 siRNA. The values are plotted after normalizing to actin (*p*<0.06).

We next tested if ITSN2 was a downstream mediator of the ING1a-induced defect in endocytosis and the subsequently generated senescence signal. When ITSN2 was knocked down in ING1a-expressing cells, we found that EGFR internalization kinetics were similar to control GFP-expressing cells (Figure 2B) and were significantly restored when compared to the ING1a-expressing A431 cells (Figure 6A). We next checked if loss of ITSN2 in ING1a-expressing cells affected the senescence phenotype. We found that senescence-associated β-gal staining was reduced significantly in these cells (Figure 6B), and they showed increased proliferation when assayed using the BrdU incorporation assay (Figure 6C). High levels of p16 were no longer induced by ING1a (Figure 6D), suggesting that ITSN2 was indeed a direct mediator of ING1a-induced senescence signal. Since we previously noted higher levels of both ING1a and ITSN2 in senescent versus low passage fibroblasts (Figure 3A), we next tested if ITSN2 knockdown in senescent cells could ameliorate any aspects of the senescent phenotype. Knockdown of endogenous ITSN2 in senescent fibroblasts resulted in ∼50% reduction of p16 levels, while ING1a and Rb levels remained unchanged (Figure 6E). The fact that p16 was not completely repressed implies that ITSN2 is a potent effector, but not the sole inducer of replicative senescence. We next wanted to check if knocking down ITSN2 in ING1a-expressing cells could restore the levels of E2F targets. As shown in Figure 6F, ITSN2 knockdown restored most of the E2F targets tested, suggesting that it is the up-regulation of ITSN2 by ING1a that attenuates growth factor signaling and Rb phosphorylation, leading to E2F inactivation.

### Inhibition of Endocytosis Induces Senescence Markers

**Figure 7:**
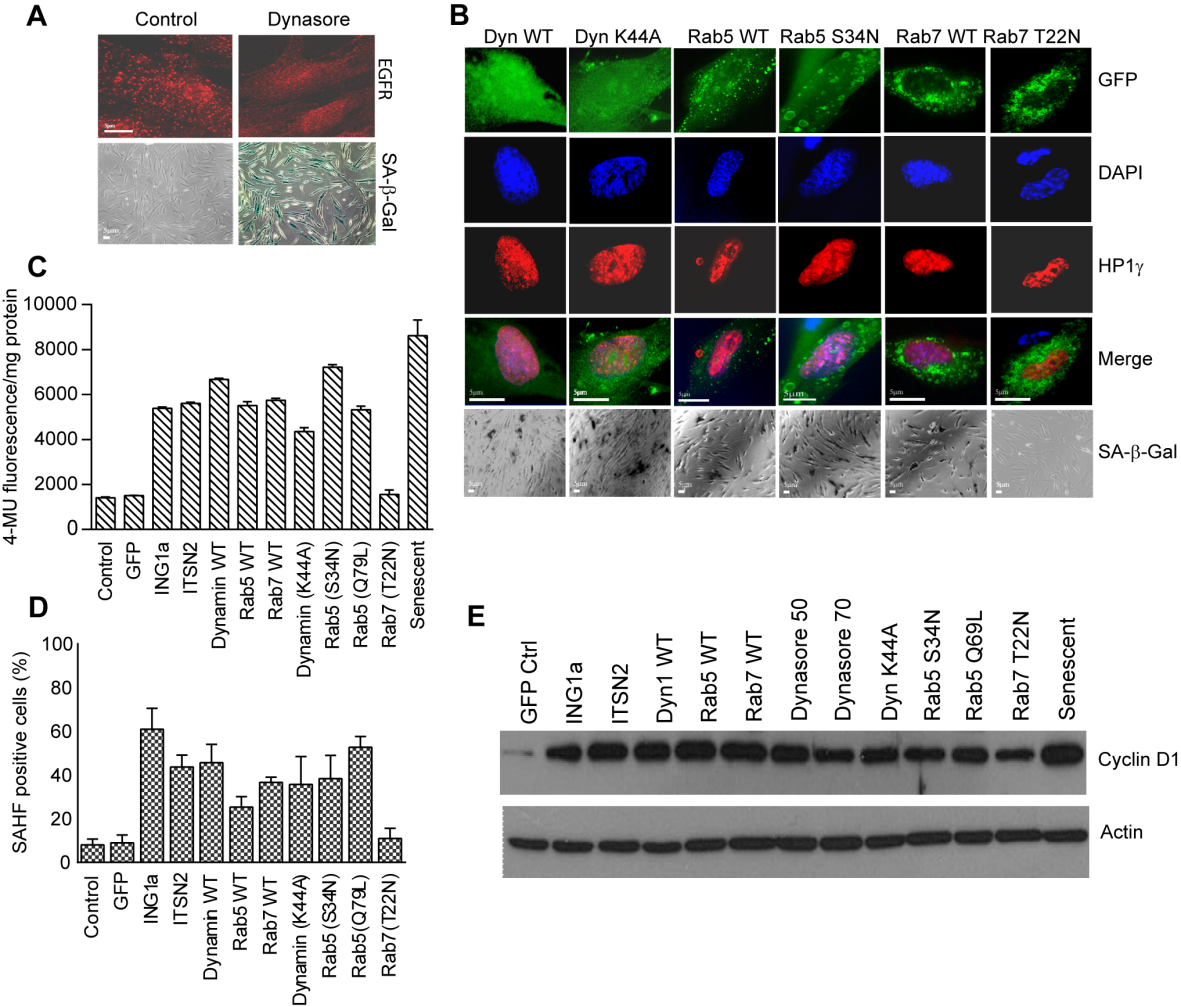
Inhibiting endocytosis leads to senescence. (A) Cells treated for 48 h with 50 µM dynasore were fixed and stained for EGFR endocytosis. Lower panels show fields of cells similarly treated and stained for the presence of SA-β-gal. (B) Constructs encoding Dynamin1 (WT & K44A mutant), Rab5 (WT & S34N), or Rab7 (WT & T22N) were transfected into young primary fibroblasts, and 24 h later, cells were fixed and stained with DAPI to visualize DNA with anti-HP1γ to visualize SAHF and with X-gal (at pH 6.0) to identify cells with SA-β-gal activity. (C) SA-β-gal activity in transfected cells was quantified using methylumbulliferryl-β-D-galactopyranoside (MUG); values were normalized to the total protein concentration of each cell lysate estimated by Lowry assay, 24 h after transfection. (D) Quantification of cells positive for the presence of SAHF from Figure 7B is plotted as histogram by cell counting. (E) Cell lysates from low passage (28 MPD) Hs68 cells transfected with the indicated constructs were immunoblotted for the cyclin D1 senescence marker. Actin was used as the loading control.

To further test whether inhibition of endocytosis by ING1a was initiating the Rb-mediated senescence signal, we asked whether inhibition of endocytosis by other methods would also lead to a senescent phenotype. Dynasore, a soluble pharmacological inhibitor of endocytosis, interferes with the GTPase activity of dynamin and thus blocks the internalization step of endocytosis [67]. Treating fibroblasts with concentrations of dynasore known to block endocytosis, resulted in cells assuming a large senescent phenotype and intense staining for SA-β-gal (Figure 7A). While Dynasore showed a clear ability to induce senescence, other nonspecific effects of this pharmacological agent cannot be ruled out. To test if inhibiting endocytosis by more specific genetic methods also induced senescence, we disrupted the stoichiometry of endocytic components, blocking endocytosis at different stages. Wild-type and dominant negative forms of Dynamin1, Rab5, and Rab7 that affect the internalization, early, and late endosomal stages of endocytosis respectively, were transfected into young fibroblasts. These cells were examined for the senescence markers: SAHF (Figure 7B and D), HP1γ (Figure 7B), SA-β-gal (as measured by direct X-gal staining in Figure 7B or by 4-MU fluorescence in Figure 7C), and cyclin D1 protein levels (Figure 7D). With the exception of the dominant negative form of Rab7 (T22N), which would be expected to interfere with the final stages of endocytosis, all wild-type and mutant endosomal proteins induced senescence phenotypes to varying degrees. Robust induction of SA-β-gal and cyclin D expression was seen in response to all constructs except Rab7 (T22N), at levels very similar to those induced by ING1a. Why expression of the wild-type but not the mutant form of Rab7 induces senescence is unknown, but it is possible that inhibition of Rab7 might have other effects on cells, since Rab7 has diverse functions, including autophagosomal formation and lysosomal biogenesisis.

## Discussion

**Figure 8:**
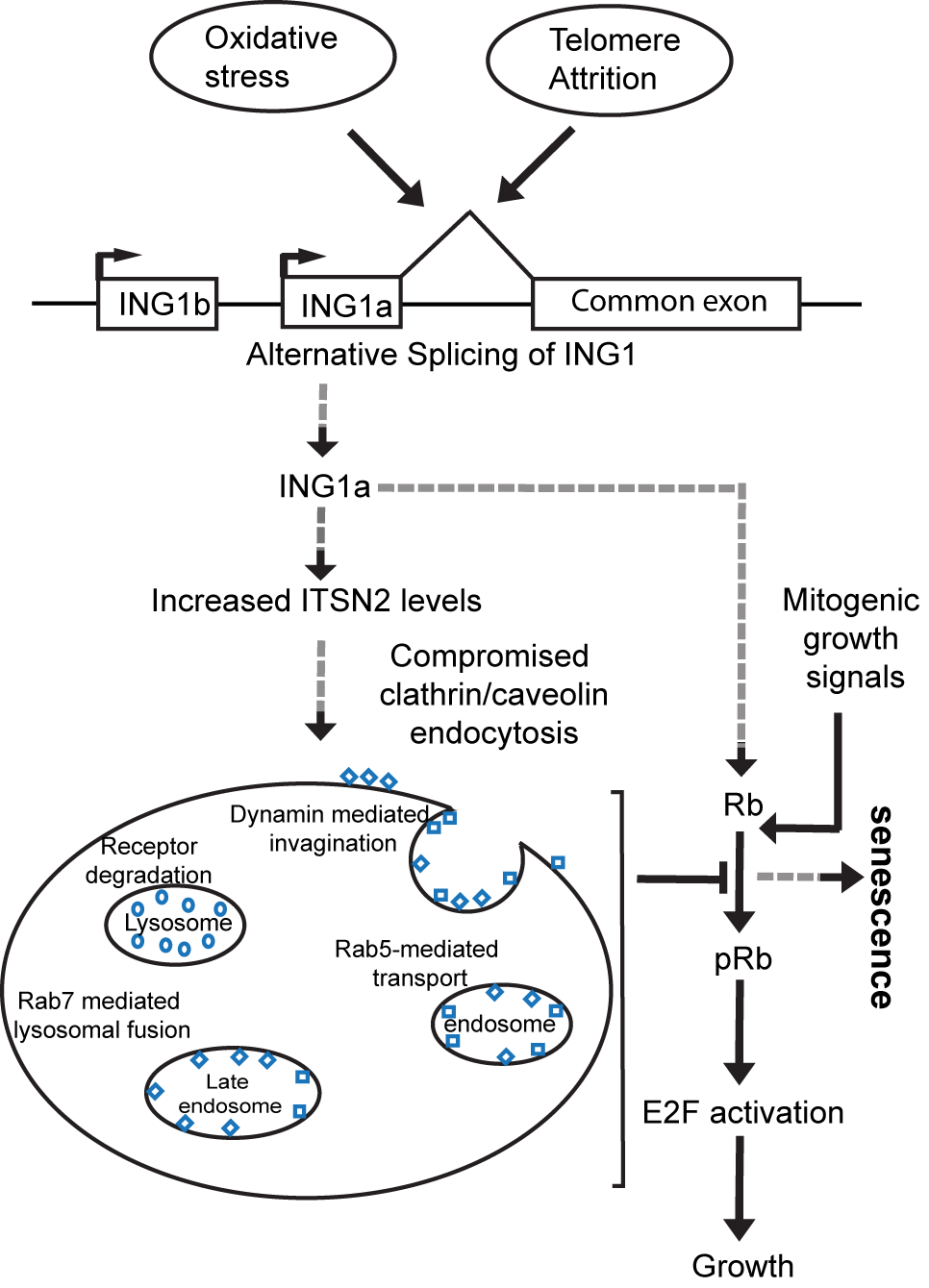
Model for ING1a-induced senescence via ITSN2 and Rb. Increased levels of ING1a, produced by alternative splicing in response to short telomeres or oxidative stress, bind and activate the promoter of ITSN2, increasing its levels. This leads to inhibition of endocytosis and a loss of signal transduction via clathrin- and caveolae-mediated endocytosis. This increases the levels of p16 and of p57^KIP2^, which inhibit Rb phosphorylation, maintaining it in its hypophosphorylated, growth-inhibitory state. This and the ITSN2-independent induction of Rb by ING1a result in the accumulation of high levels of active Rb that inhibits E2F, blocking the expression of most E2F target genes. Inability to induce transcription of growth-promoting genes inhibits cell cycle progression and, combined with Rb-induced accumulation of SAHF, results in senescence. We propose that this mechanism also acts during normal replicative senescence, since the levels of both ING1a and intersectin 2 are dramatically increased in high passage senescing cells. Dashed arrows represent the ING1a-mediated senescence pathway identified in this study.

In this study we confirm that overexpression of ING1a, which increases naturally in senescing fibroblasts, rapidly causes a form of premature senescence that mimics replicative senescence in all of the markers of senescence we examined. A disproportionate number of the genes activated by ING1a encode components of endocytosis pathways, and ITSN2, the gene showing the highest degree of induction, is a direct target of ING1a as evidenced by direct binding of ING1a to the ITSN2 promoter and rapid kinetics of ITSN2 transcriptional induction. Knockdown and knockout of ING1a reduce levels of ITSN2, consistent with ITSN2 being a *bona fide* target of ING1a. Both ING1a and ITSN2 are expressed at several-fold higher levels in senescing, compared to low passage fibroblasts, and when overexpressed, both induce p16 and p57^KIP2^ expression and senescence. Both ING1a and ITSN2 are induced in oxidative stress-induced senescent cells, suggesting that they may play roles in replicative as well as some forms of stress-induced premature senescence. This is consistent with the idea that oxidative stress contributes to replicative senescence [6]. ING1a also induces Rb by an ITSN2-independent mechanism, which the CDK inhibitors maintain in its hypophosphorylated active state. Higher levels of active Rb block the expression of E2F target genes, blocking cell cycle progression, while active Rb may also contribute to SAHF formation. How telomere attrition or oxidative stress result in increased ING1a levels by alternative isoform expression is currently unknown, but effects upon splicing of several genes have been reported as a consequence of telomere loss [22]. A model linking increased expression of ING1a to compromised endocytosis, leading to Rb activation and induction of senescence, is shown in Figure 8.

While this is the first study to report the differential expression of ITSN2 in replicative senescence, a recent report had implicated dysregution and overexpression of ITSN1 in Down Syndrome patients in an age-associated manner [68]. This is consistent with the reduced replicative capacity seen in fibroblasts from Down's patients. Our data also indicate that the ING1a–ITSN2 axis plays a causal role in senescence by dysregulating endocytosis and, consequently, signal transduction. This is fully consistent with many previous observations showing that senescing cells lose their ability to respond to exogenous mitogens, despite maintaining most growth factor receptors such as EGFR [69] and PDGFR [70], among others. Endocytosis occurs through four major pathways: clathrin-mediated endocytosis, caveolae, macropinocytosis, and phagocytosis. Among these, the clathrin-mediated and caveolar forms are responsible for receptor-mediated signal transduction found in most cells. Previous studies have implicated loss of function in both pathways as major contributors to cellular senescence [71]–[73], which is consistent with many studies demonstrating loss of sensitivity to a variety of ligands, including mitogens, in senescent cells. Many of the molecules participating in endocytosis linked to clathrin and caveolin may also function in other processes utilizing membranous structures such as autophagy, which has been implicated in the regulation of longevity through protein and organelle quality control and involves molecules such as SIRT1 [74]. In this context, it is interesting that autophagy, another cellular process that involves formation of cellular vesicles, trafficking, and lysosomal fusion, has been recently implicated in senescence as well as in tumor suppression and growth [75],[76]. Autophagy aids in cellular repair processes by degrading damaged cell components [77]. The exact mechanism by which autophagy might modulate replicative senescence is currently unclear. A recent report suggested a role for autophagy in oncogene-induced senescence programs and inhibition of autophagy-affected senescence induction in these cells. These observations are interesting because both autophagy and endocytosis are membrane-trafficking pathways that are necessary for cell survival, and these share several regulatory and effector molecules such as Rab7, Beclin-1, and Rubicon [78]–[80]. Furthermore, growth factor signaling has also been shown to regulate autophagy by Akt-mediated phosphorylation of mTOR. These observations support the role of vesicular trafficking processes and their associated signalling changes in regulating cellular life span and mediating cell senescence-associated changes.

### Concluding Remarks

The ING proteins are encoded by the multiple splicing products of five ING genes, several of which have been implicated in the regulation of cell senescence. Overexpression of many of the ING proteins blocks cell replication, induces apoptosis, or induces indices of SIPS, depending upon the cell type and experimental model employed [24], and at least one ING protein also affects the differentiation/aging of epidermal stem cells [81]. Consistent with these observations, knocking down ING1 [45] or ING2 [46] extends cell replicative lifespan, implying that both gene products contribute to transducing the senescence signal initiated by the attrition of telomeres. This is consistent with reports that ING1 accumulates in chromatin as cells senesce [42],[82]. Increased expression of ING1a and ITSN2 during replicative senescence, in a premature cell aging model (HGPS), and in response to other forms of stress suggests that premature aging syndromes such as HGPS, SIPS, and replicative senescence may have many components in common, despite being initiated by different agents. Our data also reveal that dysregulation of cytoplasmic signal transduction pathways by various means activates the Rb tumor suppressor axis through inducing Rb expression and blocking Rb inactivation, contributing to induction of the senescent phenotype.

## Materials and Methods

### Cell Culture and Transfection

Hs68 and WI38 fibroblast cell strains were obtained from the American Type Culture Collection (ATCC) and were maintained in DMEM (Lonza) supplemented with 10% fetal bovine serum (Gibco; Invitrogen) at 37°C under 5% CO_2_. Low passage young cells used were between 14 and 35 mean population doublings (MPDs) for Hs68 cells and between 20 and 30 MPDs for WI38 cells. Senescent fibroblasts were between 80 and 85 MPDs for Hs68 cells and 55 and 60 MPDs for WI38 cells. The A431 cells were maintained in high glucose DMEM supplemented with 10% FBS. Wild-type and ING1^−/−^ mouse embryonic fibroblasts were gifts from Dr. Stephen N. Jones (University of Massachusetts) and were maintained in high glucose DMEM supplemeted with 10% FBS. Plasmid and siRNA transfections in Hs68 and WI38 cells were done using lipofectamine LTX (Invitrogen) according to the manufacturer's protocol. ING1 and ITSN2 siRNA smartpools were obtained from Dharmacon, and a scrambled siRNA was used as a control.

### Microarray Experiments and Analyses

Hs68 cells, infected with either Ad-GFP or Ad-GFP-ING1a (GFP and ING1a under separate promoters), were harvested 48 h after infection and RNA was isolated. The microarray hybridization was done as described previously [31]. Briefly, the quality of RNA isolates from cells were checked using a Bioanalyzer (Agilent), and cDNA was made using indirect labelling of cDNA with the dyes Cy3 and Cy5 using a FairPlay microarray labelling kit (Stratagene) according to the manufacturer's protocol. The labelled cDNAs were then purified and combined with yeast tDNA (Stratagene) and hybridized to human oligonucleotide chips (Southern Alberta Microarray facility, University of Calgary) at 37°C for 18 h. The slides were then washed and scanned using a fluorescence laser microarray scanning device (Virtex). The data from two independent replicates and two dye reversal experiments were quantitated and normalized using Array-Pro and GeneTraffic software. Functional annotation of the genes reproducibly affected in response to ING1a, was done using Ingenuity Pathway Analysis (IPA), PANTHER, the Database for Annotation, Visualization and Integrated Discovery (DAVID), and GFINDer bioinformatic tools. Genes falling under the same functional annotation, as predicted by at least two of these bioinformatic tools, were categorized as shown in Figure 1A

### RNA Isolation and Quantitative Real-Time PCR

Total RNA from cells were isolated using TRIzol (Invitrogen) according to the manufacturer's suggestions and were reverse transcribed using an Omniscript Reverse Transcription kit (Qiagen). Gene-specific primer sequences are available from the authors upon request. Real-time PCR was carried out in triplicate using Maxima SYBR Green qPCR Mastermix (Fermentas) on an Applied Biosystems 7900HT Fast Real-time PCR system using a standard protocol. β-Actin or GAPDH were used as endogenous normalization controls. Relative fold changes were determined using the comparative threshold (CT) method.

### Immunoblotting, Immunoprecipitation, and Immunoflourescence

Total cell lysates for western blotting experiments were prepared by lysing cells in Laemmli sample buffer and boiling at 95°C for 10 min. Proteins were resolved by SDS-PAGE and then transferred to nitrocellulose membranes. We used 5% bovine serum albumin (BSA) in PBST as a blocking solution for 1 h at room temperature, and membranes were then incubated with primary antibodies for 2 h in blocking solution, washed 3 times for 10 min, and then incubated with horse-radish peroxidase (HRP)–conjugated secondary antibodies in blocking solution for 45 min at room temperature. After washing, proteins were visualized using ECL. α-EGFR, -Cyclin D1, and -GFP were from Santacruz Biotechnology; all α-phospho-antibodies were obtained from Cell Signalling; α-actin antibody was from Cell Signalling, and α-ING1 was a mouse monoclonal from the SACRI antibody facility, University of Calgary [61]. For EGFR degradation assays, cells were serum starved overnight and stimulated with 100 ng/ml of human recombinant EGF (Invitrogen) for indicated time points together with 10 µg/ml of cycloheximide (Sigma).

For immunoprecipiatation of Rb-bound E2F complex, the cells were serum starved for 24 h and then released in the presence of Ad-GFP and Ad-ING1a containing complete medium. Twenty-four hours later, the cells were lysed in lysis buffer [50 mM Tris, pH 8.0, 150 mM NaCl, 1% NP40, 10 mM EDTA, 5% glycerol, 1 mM phenylmethylsulfonylflouride (PMSF), 10 µg/ml aprotinin, and 10 µg/ml leupeptin) and immunoprecipitated with anti-Rb (BD Pharmingen). We used 30-h serum-starved cells as the quiescent cell control. The immunoprecipiated E2F was detected by using α-E2F antibody (Cell Signalling).

For immunofluorescence experiments, cells were grown on coverslips and were fixed with 4% paraformaldehyde in phosphate buffered saline (PBS) for 15 min at room temperature, permeablilized using 0.1% Triton X-100 in PBS for 5 min, and then blocked in 5% BSA in PBS for 1 h at room temperature. Cells were then incubated with primary antibodies in blocking solution for 1 h and then incubated with Alexa-488, -568, or -633 goat α-mouse or α-rabbit secondary antibodies in blocking solution for 1 h at room temperature. Cells were then washed with PBS, stained with Hoechst stain, and imaged using an LSM 510 or Axiovert 200 microscope. Immunoflourescence in Figure 2A was performed after serum starvation and EGF stimulation as described above for western blotting.

### EGFR Internalization Assay

Serum-starved A431 cells, expressing either Ad-GFP or Ad-GFP-ING1a, were stimulated with 100 ng/ml of EGF in serum-free DMEM for the indicated time points at 37°C. Cells were then washed with ice-cold PBS thrice and incubated with 0.5 mg/ml Biotin-X-NHS (Calbiochem), dissolved in borate buffer (10 mM boric acid, 150 mM NaCl, pH 8.0) for 1 h at 4°C. Biotinylation was terminated by washing twice with ice-cold 15 mM glycine in PBS and twice with ice-cold PBS. Cells were then lysed [150 mM NaCl, 50 mM Tris-HCl, pH 8.0, 1% Triton X-100, 1 mM orthovanadate, 1 mM phenylmethylsulfonyl fluoride (PMSF), 10 mg/ml aprotinin, and 10 mg/ml leupeptin] and immunoprecipitated with α-EGFR antibody (sc-03). Cell surface biotinylated EGFR was detected using horse-radish-peroxidise–conjugated streptavidin (Calbiochem).

For studying the tyrosine phosphorylation status of EGFR, A431 cells were infected with Ad-GFP/ING1a for 24 h, serum starved for 14 h, and stimulated with 100 ng/ml EGF in serum-free DMEM for the indicated time points at 37°C. After washing with ice-cold PBS, the cells were lysed in the buffer described above and immunoprecipiated with α-EGFR. The samples were resolved by 8% SDS-PAGE and blotted with phospho-tyrosine antibody (Millipore).

### Senescence-Associated β-Galactosidase Assay and Quantification

The SA-β-gal assay was carried out as described previously [8]. Briefly, cells were fixed using 3% paraformaldehyde in PBS (pH 6.0) and stained for 14–16 h at 37°C. The staining solution contained 1 mg/ml 5-bromo-4-chloro-indolyl-β-D-galactopyranoside (X-gal), 5 mM potassium ferrocyanide, 5 mM potassium ferricyanide, 150 mM NaCl, and 2 mM MgCl_2_ in PBS (pH 6.0). For quantification of SA-β-gal activity, we followed the protocol of Gary & Kindell [83] using methyl-umbulliferryl-β-D-galactopyranoside (MUG). Cells were grown in 60 mm plates and transfected with the indicated constructs using Lipofectamine LTX (Invitrogen) according to the manufacturer's protocol. After 24 h, cells were washed thrice with PBS to remove serum and other growth media components and were then lysed in a buffer containing 5 mM CHAPS, 40 mM citric acid, 40 mM sodium phosphate, 0.5 mM benzamidine, and 0.25 mM PMSF at pH 6.0. The clarified supernatant was treated with an equal volume of 2× reaction buffer at pH 6.0 (40 mM citric acid, 40 mM sodium phosphate, 300 mM NaCl, 10 mM β-mercaptoethanol, 4 mM MgCl_2_, and 1.7 mM MUG). Samples were incubated at 37°C for 2 h, and the reaction was quenched using the stop solution containing 400 mM sodium carbonate. Fluorescence was measured using a 96-well plate reader, with excitation at 360 nm and emission at 465 nm. SA-β-gal activity was normalized to the total protein concentration measured by a standard Lowry assay.

### BrdU Incorporation Assay

Hs68 cells grown on glass coverslips were treated with 30 mM 5-bromo-2′-deoxyuridine for 6 h, fixed for 20 min with acid ethanol (90% ethanol, 5% acetic acid), and then washed with PBS. Cells were then denatured in 2 M HCl for 20 min at room temperature, washed with PBS+3% BSA, and stained with 1∶500 α-BrdU antibody (Invitrogen) for 1 h at room temperature. Following a brief wash, cells were then incubated with Alexa-568 goat α-mouse secondary antibodies in PBS/BSA for 1 h at room temperature. Cells were then washed with PBS, imaged, and counted using an Axiovert 200 microscope.

### Chromatin Immunoprecipitation

ING1a binding to the promoter of ITSN2 was tested using ChIP analysis as described previously [84]. Briefly, about 3×10^8^ cells infected with either Ad-GFP or Ad-GFP-ING1a adenoviruses were cross-linked using 1% formaldehyde (Sigma) for 15 min at 37°C. Cells were harvested after quenching with 0.125 M glycine and lysed in ChIP lysis buffer (150 mM NaCl, 50 mM Tris, pH 8.0, 1% Triton X-100, 0.1% deoxycholate, 1 mM EDTA, 1 mM PMSF, 1 µg/ml aprotinin, 1 µg/ml pepstatin, and 1 µg/ml leupeptin). Extracts were sonicated eight times for 10 s each, and lysates were clarified by centrifugation at 13,000 rpm for 15 min at 4°C. We used 100 µL of this sample as input. The clarified supernatants were immunoprecipiated with either α-ING1 or with mouse IgG antisera (negative control) at 4°C for 3 h, followed by protein G Sepharose (GE Healthcare) for 1 h at 4°C. The immunoprecipitates were sequentially washed with 1 mL of ChIP lysis buffer twice, ChIP lysis buffer with 500 mM NaCl twice, and LiCl/detergent solution (10 mM Tris-HCl, pH 8.0, 250 mM LiCl, 0.5% NP-40, 0.5% sodium deoxycholate, 1 mM EDTA) twice, and finally with TE buffer (10 mM Tris and 1 mM EDTA, pH 8.0). The beads were eluted using 1% SDS and 0.1 M sodium bicarbonate solution. The eluent and the input samples were reverse-cross-linked using NaCl for 6 h at 65°C. The DNA from the samples was isolated by phenol-chloroform, followed by ethanol precipitation. Promoter binding was tested using polymerase chain reaction using primers spanning the upstream regions of ITSN2 and EPS15 start sites (primer sequences available upon request), and the primer sequences for PCNA and Cyclin A promoters were obtained from our previous report [23].

### Stress-Induced Premature Senescence

Young (low passage) fibroblasts were exposed to 70 µM t-Butyl hydroperoxide (tbhp) as oxidative stress. Tbhp, freshly diluted in DMEM containing 10% FBS, was added to cells in 1 h doses, once a day for 8 d. After the 1 h exposure to tbhp, the cells were washed twice with PBS and allowed to grow in DMEM with 10% FBS. Cells induced to senescence by doxororubicin were exposed to 100 ng/ml of the drug for 6 d. The control cells were grown in DMEM with 10% FBS without the stressing agents. Senescence induction in the stressed and control cells was checked periodically using the SA-β-gal assay using X-gal.

### Statistical Analyses

All data are expressed as mean ± standard deviation. The statistical analyses were done using *t* tests for two samples and one-way analysis of variance for differences among groups, using GraphPad Prism software. A probability of *p*<0.05 was considered to be statistically significant.

## Supporting Information

Figure S1 Ectopic expression of ITSN2 in Hs68 cells reduces EGFR endocytosis. Hs68 cells transfected with either pcDNA3.1 GFP or pcDNA3.1 GFP+ITSN2 were serum-starved overnight and stimulated with EGF for 10 min. The cells were then fixed and stained with α-EGFR to study dynamics of the endosomes. EGFR endosomes were significantly fewer in cells transfected with ITSN2 compared to the control GFP transfected cells. Cell nuclei were stained using DAPI.
(TIF)

Figure S2 Detection of ING1a isoform in mice. RNA from MEF wild-type and *ing1* ^−/−^ KO cells was isolated and reverse transcribed. These cDNAs were then analyzed for the presence of *ing1a* specific sequence using PCR. The primers were designed using sequences that are unique to the human ING1a isoform, part of which is conserved in mice. This region is located just upstream of the third exon that codes for the murine p37ing1 isoform. PCR results demonstrated the presence of this isoform in WT MEFs but not in *ing1* ^−/−^ cells.
(TIF)

Figure S3 Expression of ING1a induces ITSN2. Hs68 cells were transfected with pCI empty vector and pCI-ING1a constructs with amounts that would emulate the physiological levels of ING1a in senescent cells, to check for ITSN2 induction. We confirmed that ectopic expression of ING1a to physiological levels induced the expression of ITSN2 about 10-fold. Induction of p16 by ING1a has previously been reported and so it was used as a positive control (*p*<0.07).
(TIF)

Figure S4 EGFR endocytosis in oxidative stress- and doxorubicin-induced premature senescence. Hs68 cells were treated with 70 µM tert-butyl hydroperoxide (tbhp) or 100 ng/ml doxorubicin (dox) and were analyzed for EGFR endocytosis by immunoflourescence. We found that cells exposed to tbhp had fewer endosomes and delayed endocytosis when compared to the control cells, while dox-induced senescent cells did not show any significant difference in EGFR endocytosis.
(TIF)

Figure S5 Antibody specificity. Western blot assay using lysates of Ad-GFP- or Ad-ING1a-expressing cells to test the specificity of the antibody. This antibody is used in chromatin immunoprecipitation assays to check for binding of the ITSN2 promoter by ING1a. β-actin was used as a loading control.
(TIF)

Table S1 List of the 242 genes up-regulated by ≥1.5-fold in response to ING1a overexpression.
(PDF)

Table S2 List of the 172 genes down-regulated by ≥1.5-fold in response to ING1a overexpression.
(PDF)

## Abbreviations

Ad: adenoviral
ChIP: chromatin immunoprecipitation
DH: Dbl homology
ECL: enhanced chemiluminescence
EGFR: epidermal growth factor receptor
EH: eps homology
EPS15: epidermal growth factor receptor substrate 15
ERK: extracellular signal regulated kinase
HAT: histone acetyl transferase
HDAC: histone deacetylase
HGPS: Hutchinson Gilford Progeria Syndrome
HP1γ: heterochromatin protein1 gamma
HSP70: heat shock protein 70
ING1: INhibitor of Growth 1
ITSN2: Intersectin 2
JAK2: Janus kinase 2
KO: knock out
MAPK: mitogen-activated protein kinase
MEF: mouse embryonic fibroblast
MUG: methyl-umbulliferryl-β-D-galactopyranoside
PDGFR: platelet-derived growth factor receptor
PHD: plant homeo domain
PH: plekstrin homology
qPCR: quantitative polymerase chain reaction
Rb: retinoblastoma
SAHF: senescence-associated heterochromatic foci
SA-β-Gal: senescence-associated beta-galactosidase
SH3: Src homology
SIPS: stress-induced premature senescence
Tbhp: tert-butyl hydroperoxide
TR: transferrin receptor
WT: wildtype
X-Gal: 5-bromo-4-chloro-indolyl-β-D-galactopyranoside
