# Supplementary figures and images for: The ING1a Tumor Suppressor Regulates Endocytosis to Induce Cellular Senescence Via the Rb-E2F Pathway
Source: PLoS Biol. 2013 Mar 5;11(3):e1001502. doi: 10.1371/journal.pbio.1001502 (PMC3589274; doi:10.1371/journal.pbio.1001502)

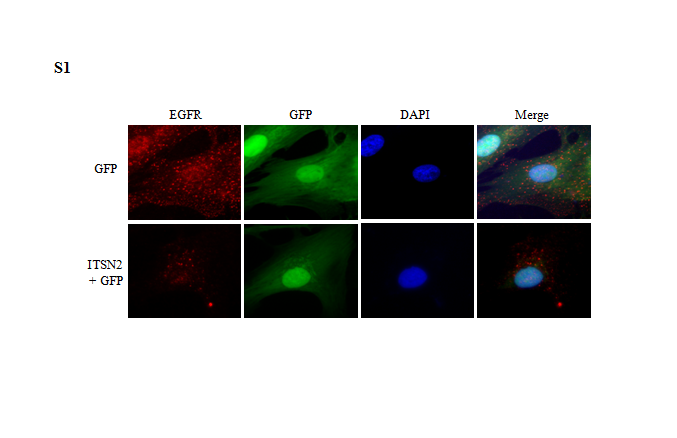

Supplement: Figure S1 — Ectopic expression of ITSN2 in Hs68 cells reduces EGFR endocytosis. Hs68 cells transfected with either pcDNA3.1 GFP or pcDNA3.1 GFP+ITSN2 were serum-starved overnight and stimulated with EGF for 10 min. The cells were then fixed and stained with α-EGFR to study dynamics of the endosomes. EGFR endosomes were significantly fewer in cells transfected with ITSN2 compared to the control GFP transfected cells. Cell nuclei were stained using DAPI. (TIF) [file pbio.1001502.s001.tif]

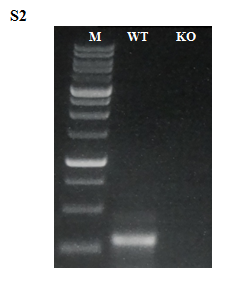

Supplement: Figure S2 — Detection of ING1a isoform in mice. RNA from MEF wild-type and ing1 −/− KO cells was isolated and reverse transcribed. These cDNAs were then analyzed for the presence of ing1a specific sequence using PCR. The primers were designed using sequences that are unique to the human ING1a isoform, part of which is conserved in mice. This region is located just upstream of the third exon that codes for the murine p37ing1 isoform. PCR results demonstrated the presence of this isoform in WT MEFs but not in ing1 −/− cells. (TIF) [file pbio.1001502.s002.tif]

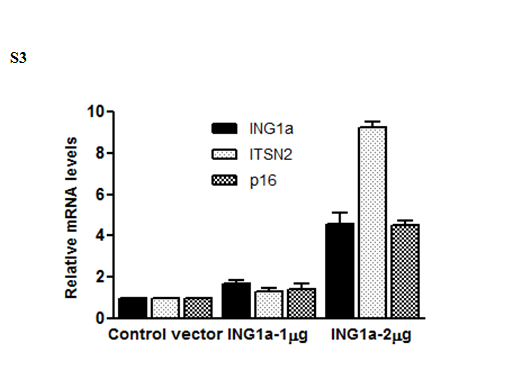

Supplement: Figure S3 — Expression of ING1a induces ITSN2. Hs68 cells were transfected with pCI empty vector and pCI-ING1a constructs with amounts that would emulate the physiological levels of ING1a in senescent cells, to check for ITSN2 induction. We confirmed that ectopic expression of ING1a to physiological levels induced the expression of ITSN2 about 10-fold. Induction of p16 by ING1a has previously been reported and so it was used as a positive control (p<0.07). (TIF) [file pbio.1001502.s003.tif]

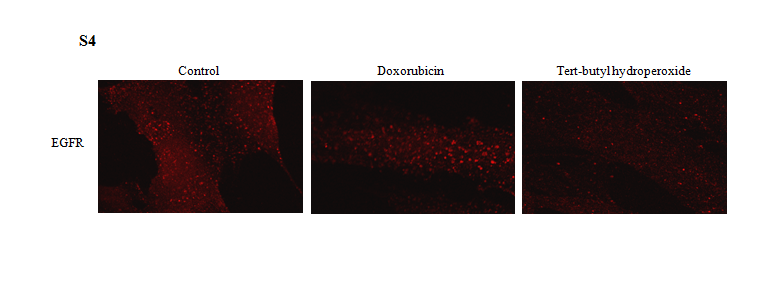

Supplement: Figure S4 — EGFR endocytosis in oxidative stress- and doxorubicin-induced premature senescence. Hs68 cells were treated with 70 µM tert-butyl hydroperoxide (tbhp) or 100 ng/ml doxorubicin (dox) and were analyzed for EGFR endocytosis by immunoflourescence. We found that cells exposed to tbhp had fewer endosomes and delayed endocytosis when compared to the control cells, while dox-induced senescent cells did not show any significant difference in EGFR endocytosis. (TIF) [file pbio.1001502.s004.tif]

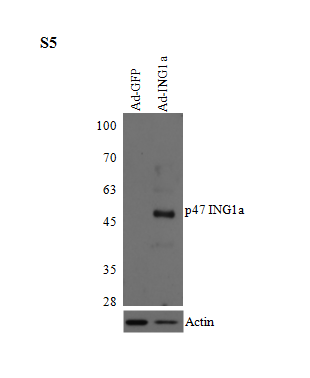

Supplement: Figure S5 — Antibody specificity. Western blot assay using lysates of Ad-GFP- or Ad-ING1a-expressing cells to test the specificity of the antibody. This antibody is used in chromatin immunoprecipitation assays to check for binding of the ITSN2 promoter by ING1a. β-actin was used as a loading control. (TIF) [file pbio.1001502.s005.tif]
