# Supplementary material for: The ING1a Tumor Suppressor Regulates Endocytosis to Induce Cellular Senescence Via the Rb-E2F Pathway
Source: PLoS Biol. 2013 Mar 5;11(3):e1001502. doi: 10.1371/journal.pbio.1001502 (PMC3589274; doi:10.1371/journal.pbio.1001502)
Supplement: Table S1 — List of the 242 genes up-regulated by ≥1.5-fold in response to ING1a overexpression. (PDF) [file pbio.1001502.s006.pdf]

Supplementary table S1: Genes upregulated ≥ 1.5 in response to ING1a over-expression

Gene ID	Fold Change	Gene Description
U61167	27.65	SH3 domain protein 1B (ITSN2)
NM_4972.3	11.27	Janus kinase 2 (tyrosine kinase)
AL117496	8.32	Hypothetical protein
D45370	8.54	adipose specific 2
D14446	8.39	fibrinogen-like 1
NM_000401	7.88	exostoses (multiple) 2
AF007149	7.45	Homo sapiens clone 23568, 23621, 23795, 23873 and 23874 mRNA sequences
M11717	7.3	heat shock 70kD protein 1
AK001117	6.06	Homo sapiens cDNA FLJ10255 fis, clone HEMBB1000852
AF026852	5.62	SCO (cytochrome oxidase deficient, yeast) homolog 1
U41492	5.34	guanine nucleotide binding protein (G protein), gamma transducing activity polypeptide 1
U69141	4.89	glutaryl-Coenzyme A dehydrogenase
Z11502	4.85	annexin A13
M21305	4.82	Human alpha satellite and satellite 3 junction DNA sequence
NM_001130	4.77	amino-terminal enhancer of split
AF182077	5.36	glioma tumor suppressor candidate region gene 1
NM_015978	4.57	putative protein-tyrosine kinase
L38518	4.68	sonic hedgehog (Drosophila) homolog
NM_006948	4.23	stress 70 protein chaperone, microsome-associated, 60kD
M93284	4.16	pancreatic lipase-related protein 2
NM_015978	4.12	Putative-protein tyrosine kinase
Z29064	3.98	epidermal growth factor receptor pathway substrate 15
U29725	3.86	mitogen-activated protein kinase 7
M21570	3.83	Human Transferrin (TF) gene
Y08639	3.82	RAR-related orphan receptor B
AK001506	3.81	DEAD/H (Asp-Glu-Ala-Asp/His) box polypeptide 20, 103kD
U80113	3.81	Human immunoglobulin heavy chain variable region (V4-31) gene, partial cds
S66407	3.76	FLT4
AF130372	3.73	serine-threonine protein kinase NKIAMRE
AJ271351	3.61	putative transcriptional regulator
U91616	3.55	nuclear factor of kappa light polypeptide gene enhancer in B-cells inhibitor, epsilon
AF081287	3.5	CTD (carboxy-terminal domain, RNA polymerase II, polypeptide A) phosphatase, subunit 1
U09278	3.45	fibroblast activation protein, alpha; seprase
AB004064	3.35	tomoregulin
AL137688	3.21	copine VI (neuronal)
Y16610	3.17	spastic paraplegia 7, paraplegin (pure and complicated autosomal recessive)
AJ005801	3.05	protein phosphatase 1B (formerly 2C), magnesium-dependent, beta isoform
AC005764	2.86	Homo sapiens chromosome 19, cosmid R31343
AC005559	2.82	Homo sapiens chromosome 19, cosmid F18382, telomeric end
U12778	2.8	acyl-Coenzyme A dehydrogenase, short/branched chain

M74558	2.8	TAL1 (SCL) interrupting locus
AF003522	2.73	delta (Drosophila)-like 1
X59841	2.71	pre-B-cell leukemia transcription factor 3
NM_006596	2.67	polymerase (DNA-directed), theta
U27699	2.63	solute carrier family 6 (neurotransmitter transporter, betaine/GABA), member 12
U27328	2.62	fucosyltransferase 3 (galactoside 3(4)-L-fucosyltransferase, Lewis blood group included)
U81234	2.61	small inducible cytokine subfamily B (Cys-X-Cys), member 6 (granulocyte chemotactic protein 2)
AB017016	2.6	brain-specific protein p25 alpha
AF050081	2.57	chromosome 16 open reading frame 3
NM_016136	2.04	adaptor-related protein complex AP-4 mu4 subunit
AJ132429	2.49	hyperpolarization activated cyclic nucleotide-gated potassium channel 4
L35318	2.48	glutamate receptor, metabotropic 2
S69873	2.47	DNA polymerase beta {107bp insertion at nucleotide 550}
D38024	2.46	Human facioscapulohumeral muscular dystrophy (FSHD) gene region, D4Z4 tandem repeat unit
L13266	2.4	glutamate receptor, ionotropic, N-methyl D-aspartate 1
AJ132099	2.4	Vanin 1
L17328	2.39	pre-T/NK cell associated protein (3Cl)
AJ133534	2.39	Rab acceptor 1 (prenylated)
M16961	2.38	alpha-2-HS-glycoprotein
M96956	2.37	teratocarcinoma-derived growth factor 3, pseudogene
AF005482	2.34	histone deacetylase 3
M55422	2.32	Krueppel-related zinc finger protein
M29204	2.31	chromosome 2 open reading frame 3
U40763	2.31	Clk-associating RS-cyclophilin
AF027299	2.28	erythrocyte membrane protein band 4.1-like 2
NM_015986	2.23	cytokine receptor-like molecule 9
L42450	2.23	pyruvate dehydrogenase kinase, isoenzyme 1
AF193339	2.21	eukaryotic translation initiation factor 2 alpha kinase 3
AB011540	2.21	low density lipoprotein receptor-related protein 4
X05232	2.18	matrix metalloproteinase 3 (stromelysin 1, progelatinase)
X62055	2.17	protein tyrosine phosphatase, non-receptor type 6
AF129756	2.14	apolipoprotein M
D31889	2.14	proteasome (prosome, macropain) 26S subunit, non-ATPase, 5
X89657	2.12	a disintegrin and metalloproteinase domain 3a (cyritestin 1)
NM_016085	2.11	apoptosis related protein APR-3
AK001306	2.11	Rho GTPase activating protein 8
L38707	2.1	diacylglycerol kinase, theta (110kD)
U48508	2.09	ryanodine receptor 1 (skeletal)
AF176812	2.08	dopamine receptor D2longer
D79987	2.04	extra spindle poles, S. cerevisiae, homolog of
U33267	2.03	glycine receptor, beta
NM_004655	2.02	axin 2 (conductin, axil)
AJ130894	2	transcription factor

D86407	1.99	apolipoprotein E receptor 2
AK000188	1.99	Homo sapiens cDNA FLJ20155 fis, clone COL08754, similar to ACSA_ECOLI ACETYL-COENZYME A
AJ224874	1.97	calcium channel, voltage-dependent, alpha 1F subunit
NM_007034	1.97	DnaJ-like heat shock protein 40
Y07847	1.97	RAS-related on chromsome 22
Y00291	1.97	retinoic acid receptor, beta
S76638	1.96	nuclear factor of kappa light polypeptide gene enhancer in B-cells 2 (p49/p100)
AJ250042	1.96	putative Rab5 GDP/GTP exchange factor homologue
AF071202	1.95	ATP-binding cassette, sub-family C (CFTR/MRP), member 4
NM_016131	1.95	RAB10, member RAS oncogene family
X97335	1.94	A kinase (PRKA) anchor protein 1
NM_016206	1.94	colon carcinoma related protein
X54489	1.92	GRO1 oncogene (melanoma growth stimulating activity, alpha)
U66464	1.92	mitogen-activated protein kinase kinase kinase kinase 1
U32849	1.9	N-myc (and STAT) interactor
NM_015984	1.9	ubiquitin C-terminal hydrolase UCH37
NM_005502	1.89	ATP-binding cassette, sub-family A (ABC1), member 1
V00488	1.89	hemoglobin, alpha 2
NM_006871	1.89	receptor-interacting serine-threonine kinase 3
AF007197	1.88	Homo sapiens mucin (MUC3) gene, partial cds
AB012853	1.88	inhibitor of growth family, member 1-like
NM_013337	1.87	translocase of inner mitochondrial membrane 22 (yeast) homolog
AF051767	1.86	GDNF family receptor alpha 3
S82362	1.86	retinoic acid receptor-beta associated open reading frame
X60957	1.85	tyrosine kinase with immunoglobulin and epidermal growth factor homology domains
AJ243274	1.84	Kruppel-like factor 12
M13509	1.84	matrix metalloproteinase 1 (interstitial collagenase)
M28215	1.84	RAB5A, member RAS oncogene family
U63090	1.83	sialyltransferase 4B (beta-galactoside alpha-2,3-sialytransferase)
NM_006129	1.82	bone morphogenetic protein 1
M16967	1.82	coagulation factor V (proaccelerin, labile factor)
AF028233	1.82	distal-less homeo box 3
K01900	1.82	interferon, alpha 8
NM_014520	1.82	MYB binding protein (P160) 1a
L29339	1.82	solute carrier family 5 (sodium/glucose cotransporter), member 1
U78556	1.81	cisplatin resistance associated
U68385	1.81	Meis (mouse) homolog 3
AF060865	1.81	zinc finger protein 205
AB016899	1.8	HGC6.1.1 protein
U44105	1.8	RAB9, member RAS oncogene family, pseudogene 1
L11239	1.79	gastrulation brain homeo box 1
AC005609	1.79	Homo sapiens chromosome 5, BAC clone 203o13 (LBNL H155), complete sequence
Z83736	1.78	H2A histone family, member E

AF097730	1.77	G-substrate
AK001111	1.76	RAB-8b protein
AF044197	1.75	B-cell-homing chemokine (ligand for Burkitt's lymphoma receptor-1)
U13896	1.75	discs, large (Drosophila) homolog 1
M12272	1.74	alcohol dehydrogenase 3 (class I), gamma polypeptide
L36055	1.74	eukaryotic translation initiation factor 4E binding protein 1
M58597	1.74	fucosyltransferase 4 (alpha (1,3) fucosyltransferase, myeloid-specific)
S81944	1.74	gamma-aminobutyric acid (GABA) A receptor, alpha 6
X75342	1.73	SHB adaptor protein (a Src homology 2 protein)
L11329	1.72	dual specificity phosphatase 2
AC004382	1.72	Homo sapiens Chromosome 16 BAC clone CIT987SK-A-152E5
U40152	1.72	origin recognition complex, subunit 1 (yeast homolog)-like
U50383	1.72	retinoic acid responsive
L11702	1.71	glycosylphosphatidylinositol specific phospholipase D1
M80563	1.71	S100 calcium-binding protein A4 (calcium protein, calvasculin, metastasin, murine placental homolog)
AL031651	1.69	transglutaminase 2 (C polypeptide, protein-glutamine-gamma-glutamyltransferase)
X87949	1.67	heat shock 70kD protein 5 (glucose-regulated protein, 78kD)
AJ131188	1.67	Homo sapiens FANCA gene, exon 10a
Y14737	1.67	immunoglobulin heavy constant gamma 3 (G3m marker)
D29992	1.67	tissue factor pathway inhibitor 2
X15183	1.66	heat shock 90kD protein 1, alpha
AF056087	1.66	secreted frizzled-related protein 1
AB023149	1.66	tolloid-like 2
U79751	1.65	basic leucine zipper nuclear factor 1 (JEM-1)
NM_016326	1.65	chemokine-like factor 3, alternatively spliced
X98337	1.65	complement factor H-related 4
NM_016204	1.65	growth differentiation factor 2
AF007147	1.65	Homo sapiens clone 23712 mRNA sequence
Z49194	1.65	POU domain, class 2, associating factor 1
M83181	1.64	5-hydroxytryptamine (serotonin) receptor 1A
U46023	1.64	chromosome X open reading frame 6
M11186	1.64	oxytocin, prepro- (neurophysin I)
Y08201	1.64	Rab geranylgeranyltransferase, beta subunit
U55853	1.64	type II Golgi membrane protein
X97324	1.63	adipose differentiation-related protein; adipophilin
D26528	1.63	DEAD/H (Asp-Glu-Ala-Asp/His) box polypeptide 7 (RNA helicase, 52kD)
AF112461	1.63	G protein-coupled receptor 57
D64007	1.63	GCN5 (general control of amino-acid synthesis, yeast, homolog)-like 1
X12784	1.63	Human col4a1 and col4a2 genes for collagen type IV
U20982	1.63	insulin-like growth factor-binding protein 4
M57230	1.63	interleukin 6 signal transducer (gp130, oncostatin M receptor)
D87467	1.62	guanine nucleotide exchange factor for Rap1

AK001797	1.62	Homo sapiens cDNA FLJ10935 fis, clone OVARC1000661
AL109959	1.62	Homo sapiens mRNA full length insert cDNA clone EUROIMAGE 1019273
AF064244	1.62	intersectin 1 (SH3 domain protein)
M83664	1.62	major histocompatibility complex, class II, DP beta 1
S52784	1.61	cystathionase (cystathionine gamma-lyase)
AF000430	1.61	dynamin 1-like
NM_015507	1.61	EGF-like-domain, multiple 6
AF131791	1.61	Homo sapiens clone 25116 mRNA sequence
S70309	1.61	NF-L
AB014554	1.61	protein tyrosine phosphatase, receptor type, f polypeptide (PTPRF), interacting protein (liprin)
X69550	1.61	Rho GDP dissociation inhibitor (GDI) alpha
L78440	1.61	signal transducer and activator of transcription 4
AF064200	1.61	UDP glycosyltransferase 2 family, polypeptide B4
AL137496	1.6	cytosolic sialic acid 9-O-acetylesterase homolog
X95876	1.6	G protein-coupled receptor 9
U86529	1.6	glutathione S-transferase zeta 1 (maleylacetoacetate isomerase)
M12529	1.59	apolipoprotein E
NM_006421	1.59	brefeldin A-inhibited guanine nucleotide-exchange protein 1
M64322	1.59	protein tyrosine phosphatase, non-receptor type 7
Y00062	1.59	protein tyrosine phosphatase, receptor type, c polypeptide
K02215	1.58	angiotensinogen
X99142	1.58	keratin, hair, basic, 6 (monilethrix)
AF054284	1.58	splicing factor 3b, subunit 1, 155kD
NM_016109	1.57	angiopoietin-related protein
L39874	1.57	dCMP deaminase
M15353	1.57	eukaryotic translation initiation factor 4E
M14648	1.57	integrin, alpha V (vitronectin receptor, alpha polypeptide, antigen CD51)
AF069250	1.56	acid-inducible phosphoprotein
AB014559	1.56	chromosome 11open reading frame 11
K02765	1.56	complement component 3
Z36715	1.56	ELK3, ETS-domain protein (SRF accessory protein 2)
U17418	1.56	parathyroid hormone receptor 1
U28424	1.56	protein-kinase, interferon-inducible double stranded RNA dependent inhibitor
L40371	1.56	thyroid hormone receptor interactor 4
X55777	1.55	H.sapiens Mahlavu hepatocellular carcinoma hhc(M) DNA
U43932	1.55	Homo sapiens connexin 26 (cx26) gene, 5' region, exon 1 and partial exon 2
Y13187	1.55	Homo sapiens dmd gene, intron 11
U72882	1.55	interferon-induced protein 35
L76661	1.55	killer cell immunoglobulin-like receptor, three domains, short cytoplasmic tail, 1
M30894	1.55	T cell receptor gamma locus
U60060	1.54	fasciculation and elongation protein zeta 1 (zygin I)
Y00371	1.54	heat shock 70kD protein 10 (HSC71)
AB021868	1.54	protein inhibitor of activated STAT3

AK001357	1.53	Homo sapiens cDNA FLJ10495 fis, clone NT2RP2000297, similar to zinc finger protein 184
AF057034	1.53	microsomal NAD+-dependent retinol dehydrogenase 4
AJ223957	1.53	pleckstrin homology, Sec7 and coiled/coil domains 3
NM_016084	1.53	ras-related protein
L08961	1.53	zona pellucida receptor tyrosine kinase, 95kD
AK000324	1.52	apobec-1 complementation factor
M86934	1.52	DNA segment, numerous copies, expressed probes (GS1 gene)
X51362	1.52	dopamine receptor D2
L40157	1.52	early endosome antigen 1, 162kD
M29893	1.52	v-ral simian leukemia viral oncogene homolog A (ras related)
Y14690	1.51	collagen, type V, alpha 2
M60278	1.51	diphtheria toxin receptor (heparin-binding epidermal growth factor-like growth factor)
U03634	1.51	lymphoid blast crisis oncogene
AF043938	1.51	muscle RAS oncogene homolog
AF060981	1.51	natural killer cell receptor, immunoglobulin superfamily member
U23070	1.51	putative transmembrane protein
AF007893	1.51	pyrimidinergic receptor P2Y, G-protein coupled, 6
Z97074	1.51	Rab9 effector p40
AB001466	1.51	signal transduction protein (SH3 containing)
X05323	1.5	antigen identified by monoclonal antibody MRC OX-2
AF032887	1.5	forkhead box O3B
AJ279254	1.5	podocin
AJ249248	1.5	putative G protein-coupled receptor
AL157430	1.5	transmembrane protein TENB2
