# Supplementary material for: The ING1a Tumor Suppressor Regulates Endocytosis to Induce Cellular Senescence Via the Rb-E2F Pathway
Source: PLoS Biol. 2013 Mar 5;11(3):e1001502. doi: 10.1371/journal.pbio.1001502 (PMC3589274; doi:10.1371/journal.pbio.1001502)
Supplement: Table S2 — List of the 172 genes down-regulated by ≥1.5-fold in response to ING1a overexpression. (PDF) [file pbio.1001502.s007.pdf]

Supplementary table S2: Genes downregulated ≥ 1.5 in response to ING1a over-expression

Gene ID	Fold Change	Gene Description
M76559	-13.03	calcium channel, voltage-dependent, alpha 2/delta subunit 1
S72487	-10.63	endothelial cell growth factor 1 (platelet-derived)
AF007548	-4.43	golgi SNAP receptor complex member 2
U49070	-3.17	protein (peptidyl-prolyl cis/trans isomerase) NIMA-interacting 1
M31994	-2.8	aldehyde dehydrogenase 1, soluble
AJ271671	-2.42	zinc/iron regulated transporter-like
AL080090	-2.38	anaphase-promoting complex 10
D45887	-2.36	calmodulin 2 (phosphorylase kinase, delta)
AF070600	-2.3	Homo sapiens clone 24703 beta-tubulin mRNA, complete cds
X52259	-2.29	Human sec oncogene for SEC protein
M57567	-2.28	ADP-ribosylation factor 5
M58028	-2.1	ubiquitin-activating enzyme E1 (A1S9T and BN75 temperature sensitivity complementing)
AL031118	-2.09	Human DNA sequence from clone 153G14 on chromosome 6p21.3-22.2.
NM_001449	-2.07	four and a half LIM domains 1
AF065241	-2.06	Homo sapiens thioredoxin delta 3 (TXN delta 3) mRNA, partial cds
X04802	-2.04	Homo sapiens UBBP2 pseudogene for ubiquitin UBB
AL031903	-2.04	Human DNA sequence from clone 1032F13 on chromosome Xq25-26.3.
X57351	-2.04	interferon induced transmembrane protein 2 (1-8D)
AF049498	-1.99	sodium channel, voltage-gated, type II, beta polypeptide
L12168	-1.98	adenylyl cyclase-associated protein
X01703	-1.98	Tubulin, alpha, brain-specific
NM_015928	-1.95	androgen-induced prostate proliferative shutoff associated protein
U39840	-1.95	hepatocyte nuclear factor 3, alpha
L22005	-1.94	cell division cycle 34
AB019565	-1.94	interferon, alpha-inducible protein (clone IFI-6-16)
U89505	-1.94	RNA binding motif protein 4
AJ276003	-1.92	GAR1 protein
D38583	-1.92	S100 calcium-binding protein A11 (calgizzarin)
U30827	-1.92	splicing factor, arginine/serine-rich 5
NM_002848	-1.91	protein tyrosine phosphatase, receptor type, O
AF006082	-1.9	ARP2 (actin-related protein 2, yeast) homolog
X87160	-1.89	sodium channel, nonvoltage-gated 1, gamma
L08048	-1.88	high-mobility group (nonhistone chromosomal) protein 1-like 10
AC004534	-1.88	mouse thiamin pyrophosphokinase homolog
L09159	-1.87	ras homolog gene family, member A
M26880	-1.87	ubiquitin C
X16609	-1.85	ankyrin 1, erythrocytic
U56637	-1.84	capping protein (actin filament) muscle Z-line, alpha 1

AL049932	-1.84	programmed cell death 4
AJ000519	-1.84	ubiquitin-conjugating enzyme E2L 3
AF131774	-1.83	Homo sapiens clone 25020 mRNA sequence
AF045581	-1.82	BRCA1 associated protein-1 (ubiquitin carboxy-terminal hydrolase)
X14445	-1.81	fibroblast growth factor 3 (murine mammary tumor virus integration site (v-int-2) oncogene homolog)
M31899	-1.8	excision repair cross-complementing rodent repair deficiency, complementation group 3
AL021938	-1.8	jumonji (mouse) homolog
Y00503	-1.8	keratin 19
AB041269	-1.78	Homo sapiens mRNA for keratin 19, partial cds, isolate:K19-141
U53445	-1.77	downregulated in ovarian cancer 1
X02152	-1.77	lactate dehydrogenase A
AB020693	-1.76	neuroendocrine-specific protein C like (foocen)
S50869	-1.76	Platelet-derived growth factor A-chain [human, Genomic, 3559 nt 2 segments]
AL050179	-1.76	tropomyosin 1 (alpha)
M11353	-1.75	H3 histone, family 3A
AL050318	-1.75	myosin regulatory light chain 2, smooth muscle isoform
AF251040	-1.75	putative nuclear protein
AF015950	-1.75	telomerase reverse transcriptase
X77584	-1.75	thioredoxin
AL117428	-1.74	DKFZP434A236 protein
U32645	-1.74	E74-like factor 4 (ets domain transcription factor)
L35240	-1.73	enigma (LIM domain protein)
AF089841	-1.73	filamin C, gamma (actin-binding protein-280)
M16660	-1.73	heat shock 90kD protein 1, beta
Y09723	-1.73	zinc finger protein 151 (pHZ-67)
AF042162	-1.72	cytochrome c oxidase subunit Va pseudogene 1
X52104	-1.72	DEAD/H (Asp-Glu-Ala-Asp/His) box polypeptide 5 (RNA helicase, 68kD)
M57892	-1.71	carbonic anhydrase VI
AB002345	-1.71	period (Drosophila) homolog 2
U22376	-1.71	v-myb avian myeloblastosis viral oncogene homolog
L06132	-1.71	voltage-dependent anion channel 1
L06133	-1.7	ATPase, Cu++ transporting, alpha polypeptide (Menkes syndrome)
Z73497	-1.7	Human DNA sequence from clone U240C2 on chromosome X
AF061736	-1.7	ubiquitin-conjugating enzyme E2L 6
Z37994	-1.69	H.sapiens alpha E-catenin pseudogene
X94754	-1.69	methionine-tRNA synthetase
M60614	-1.69	Wilms tumor associated protein
X99268	-1.68	twist (Drosophila) homolog
NM_001651	-1.67	aquaporin 5
AK000379	-1.67	asparagine synthetase
AF049703	-1.67	E74-like factor 5 (ets domain transcription factor)
AL035604	-1.67	Human DNA sequence from clone 38C16 on chromosome 6q22.33-24.1. Contains GAPD

U39318	-1.67	ubiquitin-conjugating enzyme E2D 3 (homologous to yeast UBC4/5)
NM_007038	-1.66	a disintegrin-like and metalloprotease with thrombospondin type 1 motif, 5
J04823	-1.66	cytochrome c oxidase subunit VIII
AF038847	-1.66	FK506-binding protein 6 (36kD)
K03191	-1.64	cytochrome P450, subfamily I (aromatic compound-inducible), polypeptide 1
U90920	-1.64	PTPL1-associated RhoGAP 1
X83618	-1.63	3-hydroxy-3-methylglutaryl-Coenzyme A synthase 2 (mitochondrial)
AB017908	-1.63	4F2 light chain
AF072810	-1.63	bromodomain adjacent to zinc finger domain, 1B
Z23090	-1.63	heat shock 27kD protein 1
AB022178	-1.62	calcitonin receptor
AF099664	-1.62	Cdc42 effector protein 4
X86809	-1.62	phosphoprotein enriched in astrocytes 15
AF086628	-1.62	VAMP (vesicle-associated membrane protein)-associated protein B and C
D00017	-1.61	annexin A2
U65410	-1.61	MAD2 (mitotic arrest deficient, yeast, homolog)-like 1
X85237	-1.61	splicing factor 3a, subunit 1, 120kD
Z82248	-1.61	tyrosine 3-monooxygenase/tryptophan 5-monooxygenase activation protein, eta polypeptide
X15525	-1.6	acid phosphatase 2, lysosomal
M83667	-1.6	CCAAT/enhancer binding protein (C/EBP), delta
X78924	-1.6	zinc finger protein 266
M62895	-1.59	annexin A2 pseudogene 2
AC002544	-1.59	eukaryotic translation initiation factor 3, subunit 8 (110kD)
AC004780	-1.59	hypothetical protein F17127_1
S81916	-1.59	Phosphoglycerate kinase {alternatively spliced}
AF002697	-1.58	BCL2/adenovirus E1B 19kD-interacting protein 3
AB005297	-1.58	brain-specific angiogenesis inhibitor 1
AB007977	-1.58	Homo sapiens mRNA, chromosome 1 specific transcript KIAA0508
L36149	-1.57	chemokine (C motif) XC receptor 1
M14200	-1.57	diazepam binding inhibitor (GABA receptor modulator, acyl-Coenzyme A binding protein)
D38550	-1.57	E2F transcription factor 3
U32944	-1.56	dynein, cytoplasmic, light polypeptide
S82592	-1.56	ecotropic viral integration site 1
U10860	-1.56	guanine-monophosphate synthetase
X04801	-1.56	Homo sapiens UBBP1 pseudogene for ubiquitin UBB
AL035461	-1.56	Human DNA sequence from clone 967N21 on chromosome 20p12.3-13.
AF071476	-1.56	regulator of G-protein signalling 9
K03195	-1.56	solute carrier family 2 (facilitated glucose transporter), member 1
AB004885	-1.56	tousled-like kinase 1
K00558	-1.56	tubulin, alpha, ubiquitous
NM_002390	-1.55	a disintegrin and metalloproteinase domain 11
D78014	-1.55	dihydropyrimidinase-like 3
AC004774	-1.55	distal-less homeo box 6

M22919	-1.55	myosin, light polypeptide 6, alkali, smooth muscle and non-muscle
M15990	-1.55	v-yes-1 Yamaguchi sarcoma viral oncogene homolog 1
Z46389	-1.55	vasodilator-stimulated phosphoprotein
AF100781	-1.55	WNT1 inducible signaling pathway protein 3
M11567	-1.54	angiogenin, ribonuclease, RNase A family, 5
M90356	-1.54	basic transcription factor 3-like 3
X78520	-1.54	chloride channel 3
U62437	-1.54	cholinergic receptor, nicotinic, beta polypeptide 2 (neuronal)
X92120	-1.54	DNA-damage-inducible transcript 3
AC004957	-1.54	Homo sapiens PAC clone DJ1093O17 from 7q11.23-q21
U43142	-1.54	vascular endothelial growth factor C
AL162086	-1.54	villin 2 (ezrin)
X79448	-1.53	adenosine deaminase, RNA-specific
AF062529	-1.53	nudix (nucleoside diphosphate linked moiety X)-type motif 3
S66431	-1.53	retinoblastoma-binding protein 2
X91247	-1.53	thioredoxin reductase 1
U51224	-1.53	U2 small nuclear ribonucleoprotein auxiliary factor, small subunit 1
S58544	-1.52	75 kda infertility-related sperm protein [human, testis, mRNA Partial, 2427 nt]
NM_005929	-1.52	antigen p97 (melanoma associated) identified by monoclonal antibodies 133.2 and 96.5
Y15286	-1.52	ATPase, H+ transporting, lysosomal (vacuolar proton pump) 9kD
NM_001403	-1.52	eukaryotic translation elongation factor 1 alpha 1-like 14
AB011143	-1.52	Grb2-associated binder 2
M12529	-1.51	apolipoprotein E
AJ005670	-1.51	dachshund (Drosophila) homolog
AF011466	-1.51	endothelial differentiation, lysophosphatidic acid G-protein-coupled receptor, 4
AC004908	-1.51	Homo sapiens PAC clone DJ0855D21
U31120	-1.51	interleukin 13
AF005392	-1.51	tubulin, alpha 2
X05908	-1.5	annexin A1
Z31357	-1.5	cysteine dioxygenase, type I
AL031313	-1.5	Human DNA sequence from clone 581F12 on chromosome Xq21.
NM_006447	-1.5	ubiquitin specific protease 16
